# SQST-1/p62-regulated SKN-1/Nrf mediates a phagocytic stress response via transcriptional activation of *lyst-1*/LYST

**DOI:** 10.1371/journal.pgen.1011696

**Published:** 2025-05-02

**Authors:** Aladin Elkhalil, Alec Whited, Piya Ghose

**Affiliations:** The University of Texas at Arlington, Arlington, Texas, United States of America; University of Massachusetts Medical School, UNITED STATES OF AMERICA

## Abstract

Cells may be intrinsically fated to die to sculpt tissues during development or to maintain homeostasis. Cells can also die in response to various stressors, injury or pathological conditions. Additionally, cells of the metazoan body are often highly specialized with distinct domains that differ both structurally and with respect to their neighbors. Specialized cells can also die, as in normal brain development or pathological states and their different regions may be eliminated via different programs. Clearance of different types of cell debris must be performed quickly and efficiently to prevent autoimmunity and secondary necrosis of neighboring cells. Moreover, all cells, including those programmed to die, may be subject to various stressors. Some largely unexplored questions include whether predestined cell elimination during development could be altered by stress, if adaptive stress responses exist and if polarized cells may need compartment-specific stress-adaptive programs. We leveraged Compartmentalized Cell Elimination (CCE) in the nematode *C. elegans* to explore these questions. CCE is a developmental cell death program whereby three segments of two embryonic polarized cell types are eliminated differently. We have previously employed this *in vivo* genetic system to uncover a cell compartment-specific, cell non-autonomous clearance function of the fusogen EFF-1 in phagosome closure during corpse internalization. Here, we introduce an adaptive response that serves to aid developmental phagocytosis as a part of CCE during stress. We employ a combination of forward and reverse genetics, CRISPR/Cas9 gene editing, stress response assays and advanced fluorescence microscopy. Specifically, we report that, under heat stress, the selective autophagy receptor SQST-1/p62 promotes the nuclear translocation of the oxidative stress-related transcription factor SKN-1/Nrf via negative regulation of WDR-23. This in turn allows SKN-1/Nrf to transcribe *lyst-1*/LYST (lysosomal trafficking associated gene) which subsequently promotes the phagocytic resolution of the developmentally-killed internalized cell even under stress conditions.

## Author summary

During development, cells can have many fates, one of which is to deliberately die. If a cell’s inherent ability to die is lost, unwanted cells remain, which can lead to pathologies such as abnormal brain development or cancer. Dead cell remains must also be fully and efficiently cleared away by being ingested and digested by other cells, to avoid autoimmunity. Cells that are destined to die, like any cell, can also be subject to stress, which can change cell behavior. Moreover, cells fated to die often have highly intricate shapes, such as nerve cells in the brain, and their removal may entail different strategies for different regions of the cell. In this study, we have used the pre-destined “3-in-1” death of a structurally-complex cell in the roundworm *C. elegans* as a platform to describe the genetics behind how one cell bolsters its inherent ability to consume an area of another dying cell by mounting a response to environmental stress. Specifically, we report, to our knowledge for the first time, that a well-known stress-protective protein helps turns on a gene that helps ensure that ingested parts of dead cells are fully digested and removed.

## Introduction

Programmed cell elimination is an important feature of both normal development and homeostasis [1–3] and entails both cell killing and clearance. Apoptosis is the best characterized form of developmentally programmed cell death marked by defined features and genetics [4,5]. Several other forms of non-apoptotic or non-canonical regulated cell death programs have been described in recent years [6–10]. Dying cells must subsequently be cleared efficiently via phagocytosis to prevent secondary necrosis and autoimmune consequences. During phagocytosis, cell corpses and debris are internalized following their recognition by phagocytes resulting in the formation of corpse-bearing vesicles called phagosomes that undergo a series of maturation steps [11–15]. Phagosome maturation entails the dead cell cargo-containing vesicle becoming sequentially acidified and subsequently fusing to lysosomes and the resolution of the cargo [16]. Lysosomes are membrane-surrounded acidic organelles consisting of hydrolases, membrane proteins, and numerous accessory proteins. They carry digestive enzymes and are trafficked to the phagosome vesicle allowing for ultimate digestion and resolution of the corpse/debris contained in it. While phagosome maturation has been extensively characterized [11–15], there are still poorly understood factors associated.

All cells are subject to exposure to stress and an integral part of cellular physiology is the ability to adapt and restore homeostasis to ensure normal fate and function. Cells can encounter a myriad of stressors in their lifetime including heat stress, oxidative stress, UV stress, and pathogenic stress, which they combat by mounting appropriate stress responses [17–25], which have some overlapping features that are not well understood. Stressors that can induce cell death initiation include UV [26–29], ROS [30], and heat [31].

Cells have a number of intrinsic stress responses at their disposal to maintain homeostasis and cellular quality control. These include the catabolic degrative process of autophagy [17], oxidative stress response [18], heat shock response [19,20], the Unfolded Protein Response (UPR) [21,22] and DNA damage responses [23–25]. These protective responses are known to serve to counteract the effects of stress and preserve the cell. Do these same responses play any role when cells destined to die developmentally for proper homeostasis are exposed to stress? This problem is further compounded for cells that are specialized and of highly intricate structure such as neurons. The compartments of such morphologically complex cells have vastly different microenvironments which makes it plausible that their clearance mechanisms may be different.

How external stress and the cell fate of developmentally programmed cell elimination intersect is not well-studied. Outstanding questions include: Is preserving cellular integrity the only role of a stress response? Are there bonafide stress responses to permit programmed cell elimination under stress? Are canonical molecular players for cell removal and stress response involved for the removal of the different compartments of morphologically complex cells? We considered these questions and here address *in vivo* the question of how stress and stress response may impact the intrinsic fate of specialized cells destined to be eliminated and assess how cellular homeostasis may promote proper cell elimination under duress.

**Fig 1:**
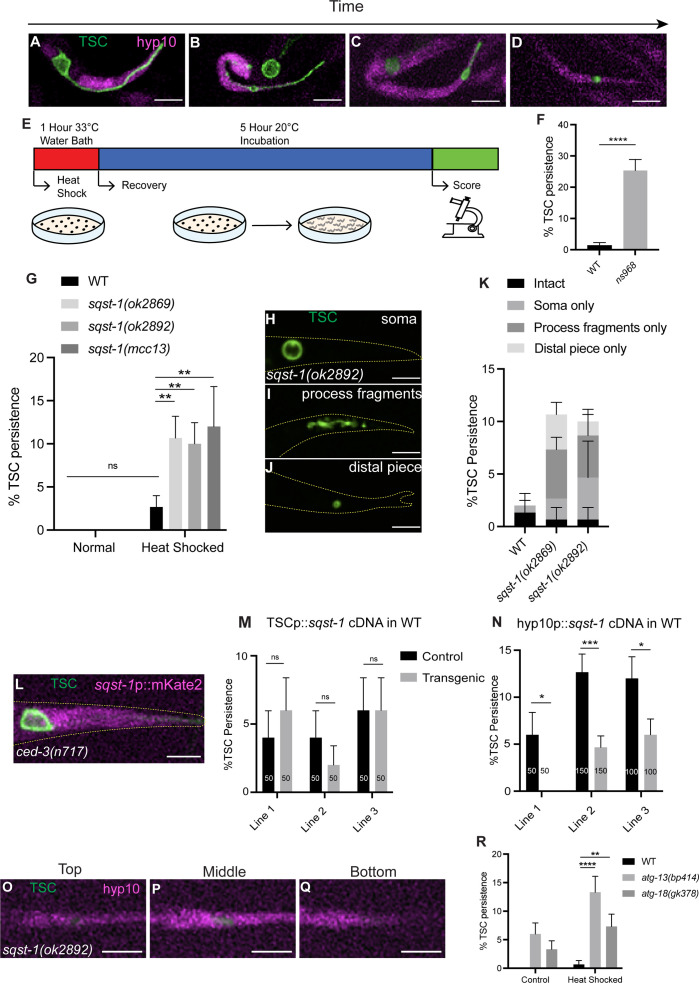
SQST-1/p62 functions in the hyp10 phagocyte to promote CCE following stress after TSC internalization. **(A-D)** CCE of tail-spike cell (TSC, green) showing hyp10 cell (magenta) adjacent to TSC process; **(A)** Intact TSC. N>5. **(B)** Rounded TSC soma, fragmenting proximal process, retracting distal process. N>5. **(C)** Soma-distal remnants. N>5. **(D)** hyp10 engulfing the TSC process remnant. N>5. **(E)** Schematic of heat stress protocol. **(F)** Quantification of *ns968* mutant CCE defects. N=50. **(G)** Quantification of TSC persistence in wild-type vs *sqst-1(-).* N>50. **(H-J)** *sqst-1(ok2892)* mutant CCE defects following heat stress. **(K)** Quantification of *sqst-1(-)* phenotype categories. **(L)** *sqst-1* reporter fluorescence in *ced-3(n717)* mutant L1 larvae, with intact TSC. N=10. **(M)** Failure of TSC-specific rescue of *sqst-1(ok2892)* defect. **(N)** hyp10-specific rescue of *sqst-1(ok2892)* CCE defect. **(O-Q)** Persisting TSC remnant internalized by hyp10 phagocyte in *sqst-1(ok2892)* mutant at the top, middle, and bottom planes of hyp10. N=10/10 animals with internalized remnants. **(R)** Quantification of TSC persistence in wild-type versus autophagy gene mutants *atg-13(bp414)* and *atg-18(gk378)* under normal and heat stress. N>50. ns (not significant) *p* > 0.05, * *p* ≤ 0.05, ** *p* ≤ 0.01, *** *p* ≤ 0.001, **** *p* ≤ 0.0001.

Previously, we have described a “tri-partite” developmental killing program for morphologically complex cells in the nematode *C. elegans* [3,10]. In this program, Compartmentalized Cell Elimination, or CCE, three segments of the *C. elegans* tail-spike epithelial cell (TSC) (Fig 1A-D) and the sex-specific CEM neurons die in different ways [3,10]. The TSC is a scaffolding epithelial cell that shapes the hyp10 tail-tip hypodermal cell (Fig 1A). During CCE, the TSC displays three degenerative morphologies – rounding soma, fragmenting proximal process, and retracting distal process (Fig 1B). The proximal process is the first to be fully removed (Fig 1C), leaving behind the soma and distal process remnants, which are cleared stochastically by different neighboring phagocytes (Fig 1D), with hyp10 acting as the process phagocyte, and an unidentified cell as the soma phagocyte. Following a forward genetic screen utilizing this system, we have previously molecularly characterized phagosome sealing, a poorly described yet critical step of phagocytosis, showing a requirement for the cell fusogen EFF-1 [10].

We employed our CCE system, specifically the TSC, to address the questions above and provide here additional insight into the process of phagocytosis in the dual context of development and stress. We describe previously unreported roles for stress-related genes in phagosome maturation. We show that, following heat stress, the selective autophagy receptor SQST-1/p62 acts within the phagocyte to stabilize the oxidative stress transcription factor SKN-1/Nrf by negatively regulating a functional analog of KEAP1 (Kelch-like ECH-associated protein 1), WDR-23, a tryptophan aspartic acid WD40-repeat protein. Mammalian Nrf proteins are key mediators of various cytoprotective responses, with the Nrf2 responses to oxidative stress the best documented [32–34]. In C. *elegans,* SKN-1/Nrf plays roles in early development [35,36] and also mediates conserved stress defense and detoxification responses and promotes longevity post-embryonically [37]. Our work shows that, in the context of CCE, SKN-1/Nrf stabilization promotes the transcription of the gene *lyst-1/*LYST (lysosomal trafficking associated), validating, *in vivo,* prior genomics studies implicating an association [38]. This regulation appears to be important for the transition from the mature phagosome to phagolysosome stage. Our study highlights phagosome maturation during cell clearance as a new context for SKN-1/Nrf function, and proposes involvement of LYST-1/LYST, a poorly characterized protein, in late phagosome-lysosome association, and highlights this association as an important control point to ensure the efficient removal of developmentally killed cells encountering stress.

## Results

### Phagocytic SQST-1/p62 promotes CCE under stress cell non-autonomously

To test the hypothesis that developmental cell death is impacted by external stress, we examined whether CCE is achieved even under stress conditions and subjected our previously employed tail-spike cell membrane-targeted GFP (TSCp::myrGFP) transgenic animals to a heat stress paradigm (Fig 1E). Contrary to our hypothesis, under normal conditions, wild type animals under stress did not show significant TSC persistence (Fig 1G). We then considered that CCE may be accomplished even under stress via a stress response that promotes intrinsic cell elimination. We therefore considered a role for cellular quality control genes. We serendipitously came upon a relevant gene following a parallel forward genetic screen with the same transgenics under normal environmental conditions. We obtained a mutant from this screen, *ns968*, with remnants of both the TSC soma and process in the first larval (L1) stage, long after the cell should be cleared (Fig 1F). Following Whole Genome Sequencing, we noted a change in the gene *sqst-1*, which encodes the homolog of mammalian p62 sequestosome, that results in a S350N change in exon 2.

p62 is a selective autophagy receptor which helps transport ubiquitinated proteins to the growing autophagosome [39–41] for their ultimate degradation. Autophagy involves the encompassing of a degradation target by an autophagosome vesicle to which lysosomes fuse, allowing for digestion by lysosomal hydrolases and destruction of the contents of the autophagosome [17]. Selective autophagy targets damaged organelles, invading pathogens and aggregated or unwanted proteins [41].

Intrigued, we attempted to confirm gene identity and tested three additional *sqst-1* loss-of-function alleles, two deletion alleles [42] and a CRISPR/Cas9-engineered allele with the same lesion as in our original mutant. However, surprisingly, none of these showed the CCE defect of *ns968* under normal conditions (Fig 1G). We then reasoned that the impact of a stress response gene mutation may only be realized under conditions of stress. As such, we subjected our *sqst-1*/p62 mutants to our heat-shock paradigm. Under these conditions, consistent with our idea, we observed robust CCE defects (Fig 1G), with a range of defects of both the soma and the process (Fig 1H-K), thus confirming gene identity.

We then asked why our original mutant *ns968* displayed a CCE defect even in the absence of stress and postulated that this may be reconciled by the presence of an additional mutation in the strain. While an intriguing possibility, we elected to continue our present study with *sqst-1*/p62 mutants under stress conditions. We do envision pursuing work on this likely second mutation in the future.

We first tested the expression of *sqst-1*/p62 using a transcriptional reporter for this gene (*sqst-1*p::mKate2). As in our previous studies [10,43] we looked at this reporter in *ced-3(n717)* loss-of-function mutants [44] bearing the TSC membrane GFP reporter. The *ced-3* null background allows us to easily visualize whether there is signal in the TSC, which survives even in mutants. We inferred expression of *sqst-1* in the hyp10 epithelial cell based on reporter fluorescence signal (Fig 1L). We have previously shown that hyp10 serves both as the animal’s tail tip and as the phagocyte for the TSC process [10]. We next performed cell-specific rescue (Fig 1M) and found that introduction of *sqst-1*/p62 in the TSC did not rescue the mutant CCE heat shock defect, consistent with the lack of fluorescence signal in the TSC. However, we observed rescue of the process phenotype by expression in hyp10 (Fig 1N). These data suggest that *sqst-1*/p62 functions in the hyp10 phagocyte to aid elimination of the TSC process cell non-autonomously. As mentioned above, we do observe CCE defects in both the TSC soma and process. Previously we have shown that the internalization of these two compartments is differentially regulated [10], with the canonical engulfment protein CED-5/Dock180 being important for TSC soma engulfment, but not process engulfment; and EFF-1 fusogen being important for phagosome sealing for the TSC process, but not the soma. However in the same study, we also found that resolution of both compartments requires SAND-1/Mon1 [10,45], which is important during phagosome (and endosome) maturation for the transition of RAB-5-positive early phagosomes to RAB-7-positive late phagosomes [45]. Based on this prior work, and because the identity of the soma phagocyte is unknown, we further proceeded with only the TSC distal process, which is phagocytosed by hyp10.

We next examined whether the TSC remnants of *sqst-1(ok2892)* animals following heat shock are internalized by hyp10 in *sqst-1*/p62 mutants. We visualized the location of these TSC process remnants (mKate2-PH) relative to hyp10 (iBlueberry) (Fig 1O-Q) and found that they were internalized. This suggests that the CCE defect following stress involves a step after corpse internalization, possibly during phagosome maturation and corpse processing.

Mammalian p62 is known to bind directly to ubiquitinated targets via its UBA domain [46]. In worms, there is a single E1 ligase, UBA-1 [47]. When exposing *uba-1(it129)* [47] to our heat shock paradigm, we observed CCE defects similar to those of our *sqst-1*/p62 mutants (S1A-D Fig). This suggests that SQST-1/p62 may act in its canonical capacity in selective autophagy together with UBA-1 to promote CCE and that these genes act in the same genetic pathway. SQST-1/p62 is a well-known conserved receptor for autophagosome formation. We tested whether the autophagy pathway is important in our paradigm. We examined *atg-13(bp414),* which bears a substitution known to lead to defects in autophagic degradation [48], as well as the null deletion mutant *atg-18(gk378)* which suppresses autophagy [49]. As in the case of *sqst-1*/p62 mutants, we saw no significant CCE defect under normal conditions, but following heat stress, we observed significant CCE defects (Fig 1R). This suggests that the stress-adaptive pathway we report may be initiated through an autophagy-mediated degradation pathway.

### SQST-1/p62 promotes SKN-1/Nrf2 function in CCE under stress

**Fig 2:**
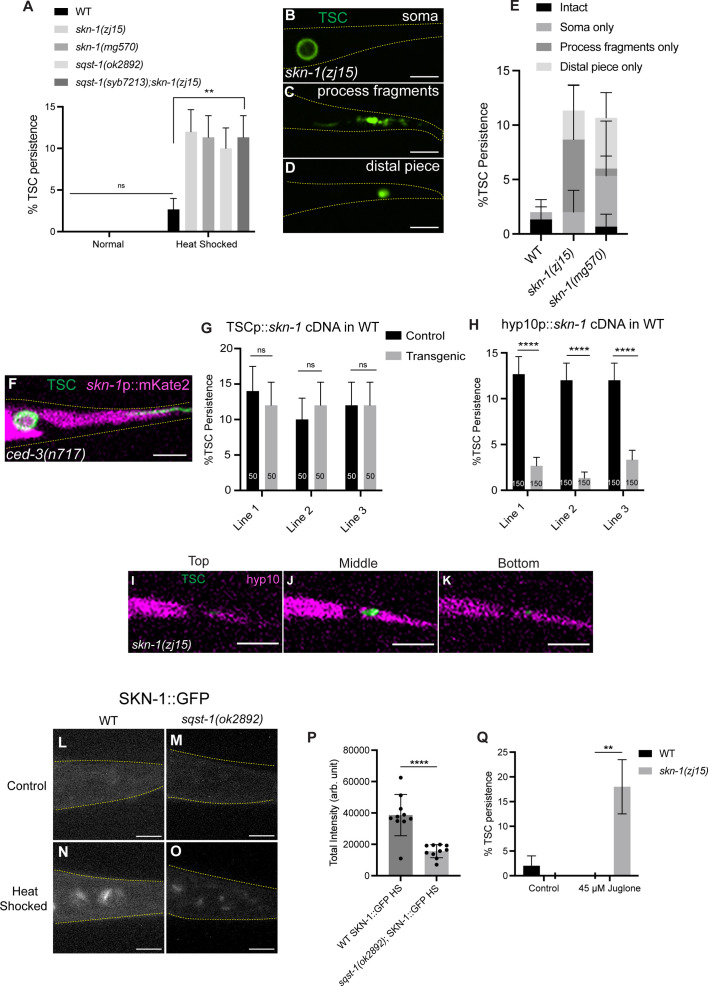
hyp10 phagocyte-specific SKN-1/Nrf2 promotes removal of internalized TSC remnants following stress in a SQST-1/p62-depedent manner. (A) Quantification of TSC persistence in wild-type vs *skn-1(-).* N > 50. (B-D) *skn-1(zj15)* mutant CCE defects following heat stress. (E) Quantification of *skn-1(-)* phenotype categories. (F) *skn-1* reporter fluorescence in *ced-3(n717)* mutant L1 larvae, with intact TSC. N = 10. (G) Failure of TSC-specific rescue of *skn-1(zj15)* defect. (H) hyp10-specific rescue of *skn-1(zj15)* defect. (I-K) Persisting TSC remnant internalized by hyp10 phagocyte in *skn-1(zj15)* mutant at the top, middle, and bottom planes of hyp10. N = 9/10 animals with internalized remnants. (L-O) Control and heat stress treated localization of SKN-1::GFP in wild-type and *sqst-1(ok2892)* mutants. N = 10. (P) Quantification of (N, O). (Q) Quantification of TSC persistence in wild-type vs *skn-1(-)* in control and following Juglone treatment (45μM). N = 50. ns (not significant) *p *> 0.05, * *p *≤ 0.05, ** *p *≤ 0.01, *** *p *≤ 0.001, **** *p *≤ 0.0001.

We next probed for the specific degradation target of SQST-1/p62. Previous studies in mammalian systems [50] have shown that mammalian SQSTM1/p62 facilitates the degradation of KEAP1, an E3 ubiquitin ligase adaptor [51–53] under oxidative stress conditions. KEAP1 is known to promote the degradation of the transcription factor Nrf2 under homeostatic conditions [54,55]. Under oxidative stress conditions, KEAP1 is slated for degradation with the help of SQSTM1/p62, thereby permitting Nrf2 to translocate to the nucleus to promote transcription of oxidative stress response genes [50]. Bearing this model in mind, we tested mutants for *skn-1*, which encodes the homolog for Nrf1 and Nrf2 in nematodes [37,56] and predicted that these mutants would phenocopy *sqst-1*/p62 mutants following heat stress. Specifically, we tested the *skn-1(zj15)* [57] and *skn-1(mg570)* [58] alleles. The *skn-1(zj15)* allele is a loss-of-function allele with an AT–GC mutation in an intron specific to *skn-1a* and *c* [57]. The *skn-1(mg570)* loss-of-function allele is reported to be relevant to proteosome dysfunction [59]. We found that both *skn-1* mutants showed similar CCE defects as those for *sqst-1*/p62 (Fig 2A-E). Moreover, a *skn-1; sqst-1* double mutant was also not additive, suggesting these genes may act in the same pathway (Fig 2A). This positions SQST-1/p62 upstream of SKN-1/Nrf.

A transcriptional reporter for *skn-1* suggests that *skn-1* is expressed in the phagocyte (Fig 2F). Moreover, *skn-1* appears to function non-autonomously in the hyp10 phagocyte based on cell-specific rescue experiments using the SKN-1a isoform (Fig 2G and 2H). *skn-1(zj15)* TSC remnants were also found to be internalized by hyp10 (Fig 2I-K). We also examined the localization of SKN-1 following heat shock and found that SKN-1b::GFP localizes to hyp10 nuclei following heat shock and that this is prevented in *sqst-1(ok2892)* mutants (Fig 2L-P). We confirmed enrichment in hyp10 nuclei using DIC optics and referencing prior literature (S2A-D Fig and [60]).

We noted that heat is not generally known as an inducer of SKN-1-dependent genes [61] and that specific isoforms of SKN-1 are known to induce other stress responses. For instance, core detoxification genes are induced by SKN-1c in response to electrophiles and pro-oxidants and Nrf2 is a transcription factor that acts in response to oxidative stress [37,56]. Proteosome genes are induced via SKN-1a in response to proteosome inhibition [59]. We asked whether the pro-phagocytic role of hyp10 SKN-1 in CCE is specific to heat. To this end, we tested for CCE defects in response to oxidative stress, and exposed animals to juglone [62,63]. Interestingly, we observed similar CCE defects in *skn-1(zj15)* mutants in presence of juglone as in heat stress (Fig 2Q). This was surprising to us given SKN-1c’s documented role in oxidative stress and our finding of a CCE heat stress response involving SKN-1a per cell-specific rescue. This suggests that both heat stress and oxidative stress and their regulation by SKN-1 are important for CCE, and highlights potentially interesting and non-canonical roles of different SKN-1 isoforms Another surprising observation in terms of SKN-1 variants is that our SKN-1::GFP localization studies are with the SKN-1b isoform, for which we were able to detect a clear signal. However, our rescue and mutant alleles are for the SKN-1a and c isoforms. One explanation for the apparent discrepancy in terms of SKN-1 isoforms is the specific cellular context of the phagocytic hyp10 during CCE (as opposed to at the whole-organism level), where SKN-1 can play a stress-coping function in response to a variety of stressors for proper corpse resolution. Future studies will further explore both the involvement of hyp10-specific SKN-1 isoforms in response to a panel of stressors and will also examine whether different genes are targeted by various SKN-1 isoforms to accomplish efficient phagocytosis, perhaps impacting different steps.

### WDR-23 negatively regulates SKN-1/Nrf and is negatively regulated by SQST-1/p62 following stress

**Fig 3:**
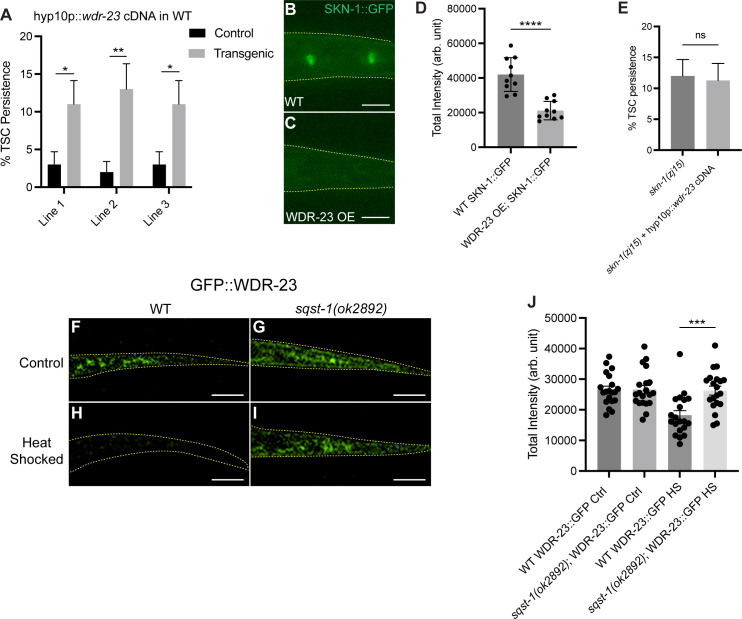
WDR-23 negatively regulates SKN-1/Nrf and is negatively regulated by SQST-1/p62 following heat stress in hyp10. **(A)** Quantification of TSC persistence in wild-type animals overexpressing hyp10-driven WDR-23. N>50. **(B-D)** Localization of SKN-1::GFP in wild-type and hyp10-specific WDR-23 expressing animals and quantification of relative fluorescence. N=10. OE: Overexpression. **(E)** Quantification of TSC persistence in *skn-1(zj15)* animals with and without hyp10-driven WDR-23. N>50. **(F-I)** CRISPR/Cas9 generated GFP tagged WDR-23 comparing fluorescence signal in wild-type control, sqst-1(ok2892) control, wild-type heat stressed, and sqst-1(ok2892) heat stressed animals. N=20. **(J)** Fluorescence intensity quantification. ns (not significant) *p* > 0.05, * *p* ≤ 0.05, ** *p* ≤ 0.01, *** *p* ≤ 0.001, **** *p* ≤ 0.0001.

We first examined the relationship between WDR-23 and SKN-1. Nrf2 is typically found in the cytoplasm bound to KEAP1, which inhibits Nrf2 under normal physiological conditions [54,55]. Following exposure to oxidative stress, KEAP1 releases Nrf2, allowing it to translocate to the nucleus and transcribe genes involved in cytoprotection [50]. While there is no known direct homolog of KEAP1 in *C. elegans,* WDR-23 is thought to degrade SKN-1/Nrf2 as a functional analog [64]. We overexpressed *wdr-23* in hyp10 in wild-type animals and found this to phenocopy the *skn-1*/Nrf2 and *sqst-1*/p62 mutants (Fig 3A). This data implicates WDR-23 as a potential KEAP1-like direct degradation target of SQST-1/p62 that negatively regulates SKN-1/Nrf2 in the context of CCE. To test whether WDR-23 acts to degrade SKN-1 in the hyp10 phagocyte, we overexpressed hyp10-specific *wdr-23* in our SKN-1b::GFP strain [65] (Fig 3B and 3C). Interestingly, we found that SKN-1::GFP levels in hyp10 decreased when *wdr-23* is overexpressed (Fig 3D), supporting the idea of WDR-23’s ability to degrade SKN-1, reminiscent of the relationship between KEAP1 and Nrf protein. Consistent with this, we found that when *wdr-23* was overexpressed in *skn-1(zj15)* mutants, we did not find an additive defect (Fig 3E). We do note that the SKN-1a variant involved on our paradigm (pre our rescue results) is known in other contexts to escape WDR-23 repression [66]. As suggested above, it is possible that the stress adaptive response we describe is independent of the nature of SKN-1 variants.

Next, we tested the idea that SQST-1/p62 negatively regulates WDR-23 during heat stress. We introduced GFP into the endogenous locus of the *wdr-23* gene via CRISPR/Cas9. We measured fluorescence intensity signal of GFP::WDR-23 in hyp10 under normal and heat stress conditions in wild type and *sqst-1(ok2892)* mutants (Fig 3F-J). In keeping with the hypothesis that SQST-1/p62 negatively regulates WDR-23 under stress, we see a decrease in GFP::WDR-23 intensity following heat stress in wild type animals, and an increase under heat stress in *sqst-1(ok2892)* animals, as well as under normal conditions in both genotypes.

### SKN-1/Nrf2 promotes *lyst-1* transcription in the hyp10 phagocyte

**Fig 4:**
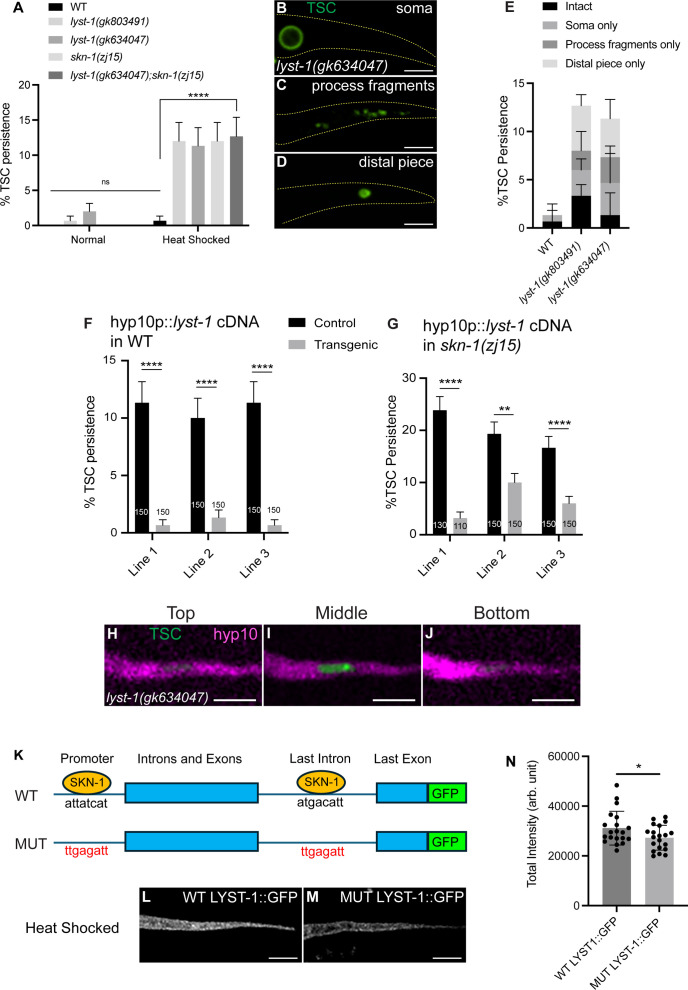
*lyst-1*/LYST promotes removal of the internalized TSC remnants following stress and is positively regulated transcriptionally by SKN-1/Nrf2. **(A)** Quantification of TSC persistence in wild-type vs *lyst-1(-).* N > 50. **(B-D)** *lyst-1(gk634047)* mutant CCE defects following heat stress. **(E)** Quantification of *lyst-1(-)* phenotype categories. **(F)** hyp10-specific rescue of *lyst-1(gk634047)* defect. **(G)** hyp10-driven *lyst-1* rescue of *skn-1(zj15)* defect. **(H-J)** Persisting TSC remnant internalized by hyp10 phagocyte in *lyst-1(gk634047)* mutant at the top, middle, and bottom planes of hyp10. N = 9/10 animals with internalized remnants. **(K)** Simplified schematic of *lyst-1* gene structure showing mutated consensus promoter and last intron binding sites of SKN-1. **(L-M)** LYST-1 GFP binding site mutants. N = 20. **(N)** Quantification of (L-M). N = 20. ns (not significant) *p *> 0.05, * *p *≤ 0.05, ** *p *≤ 0.01, *** *p *≤ 0.001, **** *p *≤ 0.0001.

We next sought to identify the transcriptional target of SKN-1/Nrf. Previous work has identified arrays of genes upregulated and downregulated in whole animals lacking *skn-1*/Nrf [38]. From this list of candidate SKN-1/Nrf targets, we tested *lyst-1*, which encodes the homolog of the lysosomal trafficking regulator LYST [67]. Mammalian LYST is a widely expressed gene encoding a protein important for membrane dynamics and intracellular trafficking of lysosomes and lysosome related organelles (LROs). However, the mechanisms underlying its function are largely unknown [68–71]. The LYST gene is conserved and while *C. elegans lyst-1* is reported to be involved in gut granule formation and other lysosome-related organelle (LRO) biogenesis [67], no direct link has been made to lysosomal trafficking to our knowledge. As above, we subjected *lyst-1/*LYST mutants harboring TSCp::myrGFP to heat stress. The *lyst-1* alleles tested were obtained from The Million Mutation Project [72] (a gift from Greg Hermann), each representing a non-sense allele that affects gut granule biogenesis [67]. We observed CCE defects akin to those in *sqst-1*/p62 and *skn-1*/Nrf mutants, with a *lyst-1; skn-1* double mutant not showing additive defects (Fig 4A-E). Our cell-specific rescue experiments suggest *lyst-1* also functions in the hyp10 phagocyte under stress conditions (Fig 4F). Additionally, we observed rescue of the *skn-1(zj15)* mutant phenotype when expressing *lyst-1* in hyp10 (Fig 4G), suggesting *lyst-1* functions downstream of SKN-1. We also found that persisting TSC remnants in *lyst-1* mutants are internalized by the hyp10 phagocyte as in the other mutants described (Fig 4H-J).

We generated a transcriptional fusion construct using the last intron of *lyst-1* (a very large intron) driving mKate2. While we did not see signal under basal conditions (S3A Fig), following heat shock, we observed reporter fluorescence in the hyp10 phagocyte (S3B Fig). Moreover, loss of *sqst-1*/p62 or *skn-1*/Nrf2 significantly reduced signal in the hyp10 phagocyte even after heat shock (S3C-E Fig).

We next sought to more directly test whether *lyst-1* is a transcriptional target of SKN-1, noting consensus sites for SKN-1 binding in both the *lyst-1* promoter region and the large intron mentioned earlier (Fig 4K). We introduced GFP via a CRISPR/Cas9 gene editing into the endogenous locus of *lyst-1* (just before the stop codon). We next mutated the SKN-1 consensus site of both the large last intron (atgacatt→ttgagatt) and promoter (attatcat→ttgagatt) (Fig 4K) of *lyst-1.* While we did see *lyst-1* reporter fluorescence following heat shock in the wild-type background, this signal was reduced in animals harboring the SKN-1 binding site mutations (Fig 4L-N). These data support the idea that SKN-1/Nrf directly targets *lyst-1* regulatory regions to promote *lyst-1* expression during stress. In support of this, SKN-1 CHIP-seq data from ModEncode do indicate binding peaks at the *lyst-1* promoter region and last intron [73,74].

### The phagocytic stress response may affect phagolysosome formation

We next asked which step following corpse internalization is affected by this new SKN-1/Nrf-dependent stress response, considering phagolysosome formation, given the involvement of *lyst-1*/LYST and its speculated association with lysosomes. To evaluate how far the L1 larval stage mutant corpses progressed in phagosome maturation, we examined markers for various phagosome stages. We first asked whether the TSC corpse is arrested in early phagosomes using the PI3P-binding probe 2X-FYVE tagged with GFP and driven by the *ced-1* promoter which is expressed in hyp10 (S4 Fig) [10]. To establish the reliability of our reporter we tested for 2X-FYVE signal around the TSC in wild-type embryos and indeed found accumulation of 2X-FYVE. We then looked at L1 larvae for our mutants (the TSC does not persist in wild-type L1 animals). We found no 2X-FYVE accumulation in *sqst-1(ok2892), skn-1(zj15)* and *lyst-1(gk634047)* mutants, suggesting that the TSC corpse, in keeping with the results above, can be successfully internalized and progress from the early to late phagosomal stage.

**Fig 5:**
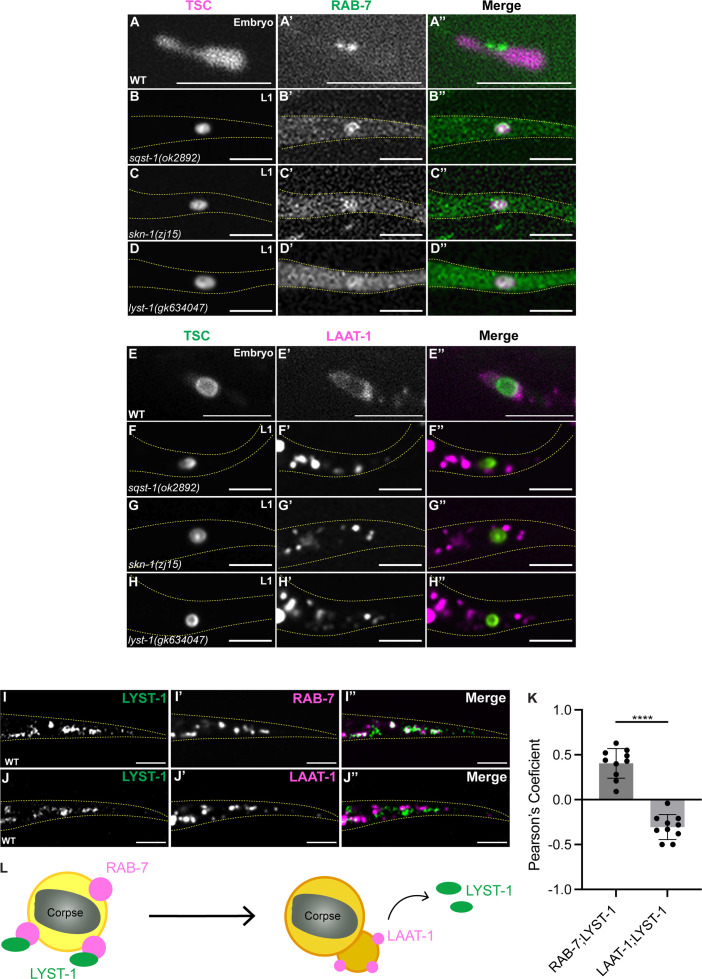
The SQST-1/SKN-1/LYST-1 stress response axis functions at the late-phagosome stage prior to phagolysosome formation. **(A-D”)** TSC remnant (magenta) localization relative to hyp10 RAB-7 (green) in wild-type, *sqst-1(ok2892)*, *skn-1(zj15)*, and *lyst-1(gk634047)* mutants. A: N = 7/7 animals with localization, B: N = 7/10 animals with localization, C: N = 9/10 animals with localization, D: N = 8/10 animals with localization. **(E-H”)** TSC remnant (green) localization relative to hyp10 LAAT-1 (magenta) in wild-type, *sqst-1(ok2892)*, *skn-1(zj15)*, and *lyst-1(gk634047)* mutants. E: N = 7/10 animals with localization, F: N = 10/10 animals lacking localization, G: N = 10/10 animals lacking localization, H: N = 9/10 animals lacking localization. **(I-I”)** Colocalization experiment for LYST-1 versus RAB-7 for hyp10 showing high degree of co-localization. Wild-type following heat stress. N = 10. **(J-J”)** Colocalization experiment for LYST-1 versus LAAT-1 for hyp10 showing lack of co-localization. Wild-type following heat stress. N = 10. **(K)** Quantification of co-localization via Pearson’s Coefficient. N = 10. (L) Schematic of LYST-1 localization in late phagosome-lysosome association.

We next sought to examine the presence of the TSC corpse in late phagosomes. The small GTPase Rab-7/RAB7, an important player in membrane trafficking [75], is known to decorate late phagosomes [76] that will subsequently fuse to incoming lysosomes [14,15], but also lysosomes, phagolysosomes [77] and late endosomes [78]. We tested hyp10-specific GFP::RAB-7 in wild-type embryos and found localization at the TSC (Fig 5A-A”). We found RAB-7 to also localize to the TSC corpse in *sqst-1(ok2892), skn-1(zj15)* and *lyst-1(gk634047)* mutants at the L1 stage (Fig 5B-D”). These data suggest that the TSC corpse can reach the late phagosomes or phagolysosomes (both of which harbor RAB-7) in mutant and wild-type backgrounds.

We made an additional surprising and intriguing observation. The RAB-7-associated TSC corpses of mutant animals were markedly different from wild-type. We acquired still images of late stage CCE progression. For mutants, we frequently found TSC corpses with the rounded morphology shown in Fig 5B–D”. However, we found it difficult to find this structure in wild-type animals, encountering instead a more bi-lobed morphology represented in Fig 5A-A”. Surprisingly, we observed RAB-7 to be specifically associated with the junction between the two lobes (7/7 animals). It is possible the step from the bi-lobed remnant to complete resolution is rapid, such that the later stage is not seen easily as CCE progresses in wild-type embryos. We speculate that RAB-7 may have a previously unreported role in non-autonomous cell scission. Future studies will address this interesting observation further.

Next, we tested the lysosomal marker LAAT-1 (hyp10-specific LAAT-1::mCherry), which would be expected to be enriched following the successful trafficking and fusion of lysosomes to the mature phagosome [76,79]. We noted that in wild-type embryos, LAAT-1 robustly surrounds the phagosome with no discernable distance in its signal and the TSC corpse (Fig 5E-E”). However, in mutant L1 larvae, LAAT-1-labeled particles were remote from the phagosome (Fig 5F-H”), with only a small fraction very close to, but not in obvious physical contact with the corpse. This suggests that *sqst-1*/p62, *skn-1*/Nrf and *lyst-1* are important for the association of lysosomes to the late phagosome.

We further investigated LYST-1’s contribution to CCE by examining its localization following heat shock relative to RAB-7 and LAAT-1 in first larval stage (L1) wild-type worms. In absence of a TSC corpse-bearing phagosome in wild-type larvae, we found RAB-7 and LAAT-1 to both be localized to what we presume to be late endosomes and endo-lysosomes. We note that the TSC corpse would not be observed at this stage due to successful phagocytosis, and that *lyst-1* would not be transcribed in absence of stress. We introduced wrmScarlett into the endogenous locus of *rab-7* in our LYST-1::GFP strain and found significant colocalization of LYST-1 with RAB-7-labelled structures (Fig 5I-I” and 5K). We introduced our hyp10-specific LAAT-1::mCherry construct into our LYST-1::GFP strain and found largely a lack of co-localization (Fig 5J-J” and 5K). Rather, LAAT-1-positive vesicles appear to exclude LYST-1.

The specific association of LYST-1 to RAB-7-positive vesicles supports the notion that LYST-1 may promote the association of lysosomes to late phagosomes. Based on these co-localization studies, we propose that LYST-1 is associating with either or both late endosomes and phagosomes, both of which are decorated with RAB-7, but not post-fusion phagolysosomes, and perhaps dissociates once fusion is accomplished.

What specific aspect of phagosome-lysosomal association is regulated by SQST-1/SKN-1/LYST-1 during stress? We examined mutants for *lmp-1*/Lamp-1, which encodes a lysosomal membrane protein important in lysosomes [80–83], and observed that these mutants phenocopied *sqst-1*/p62, *skn-1*/Nrf and *lyst-1* mutants (S5 Fig). While this data further directs us to lysosomes generally, it does not allow us to state with certainty that LYST-1 is important specifically for lysosomal fusion, tethering, or both. However, we do propose a role for LYST-1 in the transition from late phagosome to phagolysosome under stress (Fig 5L). Future studies focused on lysosomal biology and homeostasis will allow us to probe the nature of the lysosomal-phagosomal association in depth.

**Fig 6:**
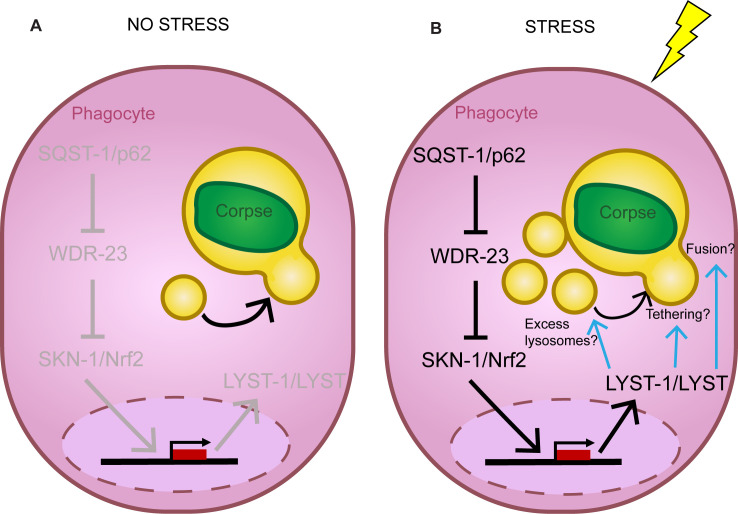
Model. SQST-1/p62 regulated SKN-1/Nrf promotes LYST-1/LYST function prior to phagolysosome formation for cell remnant resolution following stress. **(A)** Normal phagocytosis under non-stress conditions. **(B)** SQST-1/p62 promotes activity of SKN-1/Nrf2 to transcriptionally upregulate the lysosomal trafficking-associated gene *lyst-1* to ensure CCE during heat stress. This phagocytic stress response axis converges at late phagosome-lysosome association.

We speculate stress calls for a greater phagosome-lysosome association for efficient corpse resolution and we present a novel phagocytic stress response that ensures successful removal of a developmentally killed cell (Fig 6, model). We show a previously undescribed role and target for SKN-1/Nrf, as well as evidence suggesting *C. elegans* WDR-23 functions analogous to mammalian KEAP1. We also present, to our knowledge, the first evidence directly implicating *lyst-1*/LYST in phagocytosis, as well as in stress response, and highlight a step in the phagosome maturation process where it is important. Taken together, our study establishes a direct genetic link between developmental cell elimination and stress response in the context of specialized cell elimination, which may have important implications to both neurodevelopment and neurodegeneration as well as general homeostasis.

## Discussion

The interplay between stress responses and development have been described in *C. elegans* in other contexts. For example, post-embryonic studies show that stress response genes are regulated by the extracellular matrix [84] and that somatic proteostasis and stress resilience are regulated in the reproductive system as a function of the status of the embryo [85]. In other experimental systems, prior studies have linked stress and phagocytosis specifically. For example, in the murine nervous system, it has been shown that stress hormones can induce synapse phagocytosis by astrocytes [86]. On the other hand, as shown in cultured cells, oxidative stress can inhibit the phagocytosis of apoptotic cells, despite phosphatidylserine (PS) externalization [87].

Here we have employed *C. elegans* Compartmentalized Cell Elimination to demonstrate a previously unreported link between developmentally programmed cell elimination and stress response at the point of the late step of phagosome maturation and define SKN-1/Nrf as a key molecular regulator. SKN-1/Nrf has been shown to have important roles in other developmental contexts, both embryonically and post-embryonically. During early *C. elegans* embryogenesis, SKN-1/Nrf specifies development of the endoderm and mesoderm [35,36] with maternally-contributed SKN-1/Nrf functioning to establish the fate of the mesodermal precursor cell EMS [35,36]. Post-embryonic SKN-1/Nrf is well known to mediate conserved stress defense and detoxification stress responses and promote longevity [37]. Mammalian Nrf proteins have been linked to cell removal. It has been shown that overexpression of Nrf1 sensitizes cells to apoptosis on serum depletion [88]. Nrf2 is upregulated in human renal tubule cells H_2_O_2_-mediated apoptotic injury [89]. Nrf2 has also been shown to promote macrophage function following bacterial infection [90]. Our study, by describing a new role of SKN-1/Nrf in phagocytosis of a developmentally killed cell, implicates Nrf proteins at large as versatile transcription factors that assure cells achieve their intended cell removal fate. We also identify CCE as a setting to further explore the functions of SKN-1 variants and the response to various stressors. While our study implicates SKN-1 in heat stress in addition to oxidative stress, other work has linked SKN-1 with cold stress [91]. As such, there is much interest in better understanding SKN-1 as a versatile transcription factor in stress response.

By validating prior genomics work [38] that SKN-1/Nrf targets *lyst-1*/LYST *in vivo,* our study also implicates phagolysosome formation as an important point of regulation for corpse resolution following stress for a developmentally killed cell. As mentioned, LYST is a conserved protein described as important for regulation of membrane dynamics and intracellular trafficking of lysosomes and lysosome related organelles (LROs) [71]. Prior work in the nematode model describes *lyst-1*/LYST’s involvement in gut granule and other LRO biogenesis, and that *lyst-1* mutants show decreased lysosome size [67]. Otherwise, this protein remains poorly characterized. Mutations in the human LYST gene have been implicated in different diseases, most notably the autosomal recessive immunodeficiency disease Chediak-Higashi syndrome [71,92–101], marked by enlarged lysosomes and LROs [102]. An *in vivo* disease model does exist in the form of the *Lyst*-mutant ‘beige’ mouse [103]. Interestingly, in an immune context, work on macrophages suggests that *Lyst* mutations impair trafficking of the phagolysosome during the removal of bacteria [104], and LYST is important for phagosome maturation as it is required for recruitment of Rab7 during late stage endolysosomal maturation [105]. However, direct links of LYST proteins to cell corpse phagocytosis and development have not been made. In our model, we implicate a *C. elegans* LYST protein in the regulation of phagocytosis as part of a developmental cell removal program potentiated by a stress response mediated by SKN-1/Nrf. Further characterization of LYSTs and Nrfs in the context of cell corpse/debris resolution holds promise in the treatment of developmental diseases involving defects in cell elimination, as well as for other diseases marked by abnormal lysosome size and quantity. Despite the descriptive name for LYST as a lysosomal trafficking protein, LYST-1’s specific contribution to phagosome-lysosome association (fusion/transport/tethering) remains an open question. Interestingly, we observe LYST-1 at RAB-7-positive vesicles other than the phagosome (perhaps endosomes), and also LYST-1 is excluded from LAAT-1 positive structures. This motivates exploring the role of LYST-1 in the process of endocytosis following stress. A particularly intriguing observation from this study is the unexpected localization of RAB-7 in wild-type embryos during the normal progression of CCE. The localization of hyp10 phagocyte-specific RAB-7 between the two lobes of the TSC process as it degrades suggests that RAB-7 may play a new role in cell scission which will be investigated in our future studies.

Our work also implicates *C. elegans* WDR-23 in a new setting and identifies new regulation. In humans, Keap1 is the most highly studied regulator of Nrf2, negatively regulating its protein levels and activity [54,55]. No direct homolog for Keap1 is known in *C. elegans* [64]. Instead, WDR-23 has been shown to control SKN-1/Nrf [106]. Keap1 and WDR-23 are not similar in structure and their similarities are only at the mechanistic level. Nonetheless, the human genome has retained the WDR-23 homolog WDR23, despite the presence of Keap1. It has been shown that WDR23 can act as an alternative mode of regulation for Nrf2 to Keap1 [64], for example in the nervous system [107]. This has been proposed to be highly relevant to cancer biology, as cancer therapy is often associated with impairments in Keap1-dependent regulation of Nrf2 [108]. Here we present evidence supporting the idea that *C. elegans* WDR-23 acts as a functional analog to Keap1. Interestingly, while p62 has been shown to negatively regulate Keap1 [50], an involvement in WDR23 regulation has not yet been demonstrated. Our data implicates *sqst-1*/p62 as a negative regulator of WDR-23. Our findings together with the identification of additional roles and regulators of WDR-23 could thus be of therapeutic value.

Our study identifies a phagocytic stress response. In the context of CCE, the stress-exposed dying/dead cell may need additional mechanisms in place to be cleared, and it will be interesting to learn what aspect of corpse resolution the SQST-1/SKN-1/LYST-1 axis regulates. It will also be interesting to tease apart whether the phagocytic response is due to the perception of external stress of the unideal yet internalized corpse. It is also possible that another phagosome maturation step is weakened under stress and LYST-1 function must compensate. There is also a temporal consideration, as the phagocyte may be primed ahead of time by the environmental stress and then subsequently mounts its response to the corpse. In addition, whether clearance following other types of cell death, such as apoptosis or Linker cell-type death [3,9], are also similarly impacted by stress, and whether other steps of phagocytosis are regulated via specific stress responses will be interesting avenues to explore. Future screens combining the powerful CCE system with stress conditions may shed light on these and other open questions stemming from this study.

## Materials and Methods

### *C. elegans* methods

*C. elegans* strains were cultured using standard methods on *E. coli* OP50 and grown at 20ºC. Wild-type animals were the Bristol N2 subspecies. For most TSC experiments, one of two integrated reporters were used: *nsIs435* or *nsIs685.* For hyp10 experiments, *nsIs836* was used. Integration of extrachromosomal arrays was performed using UV and trioxsalen (T2137, Sigma). Animals were scored at 20ºC.

### Stress assays

#### Heat shock assay.

For most experiments, embryos were subjected to heat shock in a water bath at 33ºC for one hour and scored at the L1 stage following a recovery at 20ºC for five hours, with the exception of *lyst-1* reporter fluorescence experiments, for which animals were subjected to heat shock at 37ºC for one hour and scored immediately after.

#### Juglone drug treatments.

Embryos were treated in 45µM of 5-hydroxy-p-naphthoquinone (juglone) diluted in M9 medium (42 mM Na_2_HPO_4_, 22 mM KH_2_PO_4_, 8.6 mM NaCl and 1 mM MgSO_4_) overnight and scored for TSC persistence the following day at the L1 stage. Control animals were treated with M9 medium alone overnight. Juglone stock solution of 6mM was made by dissolving 0.025g of juglone in 23.93 mL of 100% ethanol, stirred in the dark for an hour. 45µM dilution was made by dissolving 45µL of stock in 6mL of M9 medium. Diluted solution was immediately exposed to embryos as to not lose toxicity [62,63].

### Imaging

Images were collected on a Nikon TI2-E Inverted microscope using a CFI60 Plan Apochromat Lambda 60x Oil Immersion Objective Lens, N.A. 1.4 (Nikon) and a Yokogawa W1 Dual Cam Spinning Disk Confocal. Images were acquired using NIS-Elements Advanced Research Package. For still embryo imaging, embryos were anesthetized using 0.5M sodium azide. Larvae were paralyzed with 10mM sodium azide. DIC Widefield imaging was performed on a Carl Zeiss Axio Imager.M2 microscope with 63X oil immersion lens. Super-resolution images were taken using a VTiSIM Super resolution Live Cell Confocal Imaging System.

### Image quantifications

#### Colocalization analysis.

Pearson’s correlation coefficients for GFP and mCherry, and for GFP and wrmScarlett signals were calculated (ImageJ, Coloc2) [109]. The relevant cell region (hyp10) for 10 animals for each genotype were assessed.

#### Quantification of fluorescence intensity.

Sum intensity projections of fluorescent reporters in relevant cell regions (hyp10) were generated by following DIC in ImageJ software, and GFP intensity was then calculated. Corrected Total Cell Fluorescence (CTCF) was calculated using Microsoft Excel and graphed using GraphPad Prism. Statistical significance was determined by unpaired two-tailed t-test for comparison between wild type and mutant animals.

#### Quantifications of CCE defects.

TSC death defects were scored at the L1 stage. Animals were mounted on slides on 2% agarose-M9 pads, paralyzed with 10mM sodium azide, and examined on a Zeiss Axio-Scope A1. The persisting TSC was identified by fluorescence based on its location and morphology.

##### Worm strains used in this study

LGI- *wdr-23(mcc37)*

LGII- *rab-7(mcc38)*

LGIII- *atg-13(bp414)*

LGIV- *sqst-1(ok2869), sqst-1(ok2892)*, *sqst-1(mcc13), skn-1(zj15), skn-1(mg570), ced-3(n717), uba-1(it129)*

LGV- *atg-18(gk378)*

LGX- *lyst-1(gk634047), lyst-1(gk803491)*, *lyst-1(syb8801), lyst-1(syb8801 syb9206 syb9268), lmp-1(nr2045)*

### Plasmids and transgenics

Plasmids were generated via Gibson cloning. Primer sequences and information on the construction of plasmids used in this study are provided in S1 Table. The full list of transgenes is described in S2 Table. The full length or fragment of the *aff-1* promoter was used to label the TSC. The *eff-1* promoter was used to label hyp10.

### CRISPR Cas9 genome editing

The allele of *sqst-1(mcc13)* was made with a mutation resulting in a S350N change in exon 2 (the same site as *ns968*). Mutants were generated using a co-injection strategy [110]. Guide crRNA, repair single-stranded DNA oligos, tracrRNA, and buffers were ordered from IDT. Guide crRNA used to generate *sqst-1(mcc13)* was 5’ GATCATTGAACGCTCGACCA -3’.

The allele of *lyst-1*, syb8801[lyst-1::GFP] syb9206[lyst-1 last intron, (A11985bpT,C11989bpG] syb9268[lyst-1 promoter, g.-133_-139attatca > ttgagat] was generated by Suny Biotech (Suzhou, Jiangsu, China 215028).

The allele of *wdr-23(mcc37)* was made by introducing GFP just upstream of the start of the wdr-23 gene via CRISPR/Cas9 to generally endogenously N-terminally tagged GFP::WDR-23. Mutants were generated using a co-injection strategy [110]. Guide crRNA, repair single-stranded DNA oligos, tracrRNA, and buffers were ordered from IDT. Guide crRNA used to generate *wdr-23(mcc37)* was 5’ GGTTGAATGTGAATGAGTGA-3’.

The allele of *rab-7(mcc38)* was made by introducing wrmScarlett just upstream of the start of the rab-7 gene via CRISPR/Cas9 to generally endogenously N-terminally tagged mScarlett:: RAB-7. Mutants were generated using a co-injection strategy [110]. Guide crRNA, repair single-stranded DNA oligos, tracrRNA, and buffers were ordered from IDT. Guide crRNA used to generate *rab-7(mcc38)* was 5’ AATGTCGGGAACCAGAAAGA-3’.

### Statistics

Sample sizes and statistics were based on previous studies of CCE and the TSC [10,43]. Independent transgenic lines were treated as independent experiments. An unpaired two-tailed *t-*test was used for all persisting TSC quantifications, fluorescent intensities, and colocalization analyses (GraphPad Prism). For all figures, mean ± standard error of the mean (s.e.m.) is represented.

## Supporting information

S1 Fig *uba-1*/UBA1 mutants show similar CCE defects to sqst-1/p62 mutants following stress. **(A-C)** *uba-1(it129)* mutant CCE defects following heat stress. **(D)** Quantification of TSC persistence in wild-type vs *uba-1(it129).* N > 50. ns (not significant) *p *> 0.05, * *p *≤ 0.05, ** *p *≤ 0.01, *** *p *≤ 0.001, **** *p *≤ 0.0001.
(TIF)

S2 Fig Wide field fluorescence images for SKN-1::GFP in (A, A’) wild-type control, (B-B’) *sqst-1(ok2892)* control, (C-C’) wild-type heat shocked and (D-D’) *sqst-1(ok2892)* heat shocked showing hyp10 nuclei via DIC. N = 10 for all.
(TIF)

S3 Fig *lyst-1*/LYST is expressed in hyp10 following stress and that expression is reduced in *sqst-1*/p62 and skn-1/Nrf mutants. **(A)** Wild-type animal showing no expression of *lyst-*1/LYST in hyp10 under basal conditions. N = 10. **(B)** Wild-type animal showing expression of *lyst-1*/LYST in hyp10 following heat stress. N = 10. **(C)** *sqst-1(ok2892)* mutant showing no expression of *lyst-1*/LYST in hyp10 following heat stress. N = 10. **(D)** *skn-1(zj15)* mutant showing no expression of *lyst-1*/LYST in hyp10 following heat stress. N = 10. **(E)** Quantification of B-D. ns (not significant) *p *> 0.05, * *p *≤ 0.05, ** *p *≤ 0.01, *** *p *≤ 0.001, **** *p *≤ 0.0001.
(TIF)

S4 Fig hyp10 *(ced-1p)* 2X-FYVE::GFP localization in (A-A”) wild-type, N = 1, (B-B”) *sqst-1(ok2892)*, N = 9/10 animals lacking localization, (C-C”) *skn-1(zj15)*, N = 9/10 animals lacking localization, and (D-D”) *lyst-1(gk634047)*, N = 9/10 animals lacking localization, relative to the TSC corpse in (A) the 3-fold embryo and (B-D”) L1 larvae. (TIF)

S5 Fig (A) Quantification of TSC persistence in wild-type vs *lmp-1(nr2045).* N > 50. **(B-D)** *lmp-1(nr2045)* mutant CCE defects following heat stress. **(E)** Quantification of *lmp-1(-)* phenotype categories. **(F)** hyp10-specific rescue of *lmp-1(nr2045)* defect. ns (not significant) *p *> 0.05, * *p *≤ 0.05, ** *p *≤ 0.01, *** *p *≤ 0.001, **** *p *≤ 0.0001.
(TIF)

S1 Data Numerical data underlying graphs in main and supplementary figures. (XLSX)

S1 Table Plasmids used in this study. (PDF)

S2 Table List of transgenes and strains. (PDF)

S1 Movie *sqst-1(ok2892)* mutant remnants appear internalized by hyp10. (AVI)

S2 Movie *skn-1(zj15)* mutant remnants appear internalized by hyp10. (AVI)

S3 Movie *lyst-1(gk634047)* mutant remnants appear internalized by hyp10. (AVI)

S4 Movie 2X-FYVE::GFP localization around degrading distal fragment in wild-type. (AVI)

S5 Movie Lack of 2X-FYVE::GFP localization around remnant of *sqst-1(ok2892)* mutant. (AVI)

S6 Movie Lack of 2X-FYVE::GFP localization around remnant of *skn-1(zj15)* mutant. (AVI)

S7 Movie Lack of 2X-FYVE::GFP localization around remnant of *lyst-1(gk634047)* mutant. (AVI)

S8 Movie RAB-7::GFP localization around degrading distal fragment in wild-type. (AVI)

S9 Movie RAB-7::GFP localization around remnant of *sqst-1(ok2892)* mutant. (AVI)

S10 Movie RAB-7::GFP localization around remnant of *skn-1(zj15)* mutant. (AVI)

S11 Movie RAB-7::GFP localization around remnant of *lyst-1(gk634047)* mutant. (AVI)

S12 Movie LAAT-1::mCherry showing fused lysosomes to degrading distal fragment in wild-type. (AVI)

S13 Movie LAAT-1::mCherry showing lysosomes unfused to remnant of *sqst-1(ok2892)* mutant. (AVI)

S14 Movie LAAT-1::mCherry showing lysosomes unfused to remnant of *skn-1(zj15)* mutant. (AVI)

S15 Movie LAAT-1::mCherry showing lysosomes unfused to remnant of *lyst-1(gk634047)* mutant. (AVI)

S16 Movie LYST-1::GFP colocalization with wrmScarlett::RAB-7 in wild-type following heat stress. (AVI)

S17 Movie Lack of LYST-1::GFP colocalization with LAAT-1::mCherry in wild-type following heat stress. (AVI)

## References

[1] Ellis RE, Yuan JY, Horvitz HR. Mechanisms and functions of cell death. Annu Rev Cell Biol. 1991;7:663–98. doi: 10.1146/annurev.cb.07.110191.003311 1809356

[2] Fuchs Y, Steller H. Programmed cell death in animal development and disease. Cell. 2011;147(4):742–58. doi: 10.1016/j.cell.2011.10.033 22078876 PMC4511103

[3] Ghose P, Shaham S. Cell death in animal development. Development. 2020;147(14). 10.1242/dev.191882 PMC7390631 32709690

[4] Clarke P. Developmental cell death: morphological diversity and multiple mechanisms. Anat Embryol. 1990;181(3):195–213. 10.1007/BF00174615 2186664

[5] Kerr JF, Wyllie AH, Currie AR. Apoptosis: a basic biological phenomenon with wide-ranging implications in tissue kinetics. Br J Cancer. 1972;26(4):239–57. doi: 10.1038/bjc.1972.33 4561027 PMC2008650

[6] Abraham MC, Lu Y, Shaham S. A morphologically conserved nonapoptotic program promotes linker cell death in Caenorhabditis elegans. Dev Cell. 2007;12(1):73–86. doi: 10.1016/j.devcel.2006.11.012 17199042

[7] Berry DL, Baehrecke EH. Growth arrest and autophagy are required for salivary gland cell degradation in Drosophila. Cell. 2007;131(6):1137–48. doi: 10.1016/j.cell.2007.10.048 18083103 PMC2180345

[8] Denton D, Shravage B, Simin R, Mills K, Berry DL, Baehrecke EH, et al. Autophagy, not apoptosis, is essential for midgut cell death in Drosophila. Curr Biol. 2009;19(20):1741–6. doi: 10.1016/j.cub.2009.08.042 19818615 PMC2783269

[9] Kutscher LM, Shaham S. Non-apoptotic cell death in animal development. Cell Death Differ. 2017;24(8):1326–36. doi: 10.1038/cdd.2017.20 28211869 PMC5520451

[10] Ghose P, Rashid A, Insley P, Trivedi M, Shah P, Singhal A, et al. EFF-1 fusogen promotes phagosome sealing during cell process clearance in Caenorhabditis elegans. Nat Cell Biol. 2018;20(4):393–9. doi: 10.1038/s41556-018-0068-5 29556089 PMC5876135

[11] Cheng S, Wang K, Zou W, Miao R, Huang Y, Wang H, et al. PtdIns(4,5)P₂ and PtdIns3P coordinate to regulate phagosomal sealing for apoptotic cell clearance. J Cell Biol. 2015;210(3):485–502. doi: 10.1083/jcb.201501038 26240185 PMC4523610

[12] Liu J, Li M, Li L, Chen S, Wang X. Ubiquitination of the PI3-kinase VPS-34 promotes VPS-34 stability and phagosome maturation. J Cell Biol. 2018;217(1):347–60. doi: 10.1083/jcb.201705116 29092895 PMC5748982

[13] Haley R, Wang Y, Zhou Z. The small GTPase RAB-35 defines a third pathway that is required for the recognition and degradation of apoptotic cells. PLoS Genet. 2018;14(8):e1007558. doi: 10.1371/journal.pgen.1007558 30138370 PMC6107108

[14] Kinchen JM, Doukoumetzidis K, Almendinger J, Stergiou L, Tosello-Trampont A, Sifri CD, et al. A pathway for phagosome maturation during engulfment of apoptotic cells. Nat Cell Biol. 2008;10(5):556–66. 18425118 10.1038/ncb1718 PMC2851549

[15] Kinchen JM, Ravichandran KS. Identification of two evolutionarily conserved genes regulating processing of engulfed apoptotic cells. Nature. 2010;464(7289):778–82. doi: 10.1038/nature08853 20305638 PMC2901565

[16] Kinchen JM, Ravichandran KS. Phagosome maturation: going through the acid test. Nat Rev Mol Cell Biol. 2008;9(10):781–95. doi: 10.1038/nrm2515 18813294 PMC2908392

[17] Glick D, Barth S, Macleod KF. Autophagy: cellular and molecular mechanisms. J Pathol. 2010;221(1):3–12. doi: 10.1002/path.2697 20225336 PMC2990190

[18] Thorpe GW, Fong CS, Alic N, Higgins VJ, Dawes IW. Cells have distinct mechanisms to maintain protection against different reactive oxygen species: oxidative-stress-response genes. Proc Natl Acad Sci U S A. 2004;101(17):6564–9. doi: 10.1073/pnas.0305888101 15087496 PMC404085

[19] Schlesinger MJ. How the cell copes with stress and the function of heat shock proteins. Pediatr Res. 1994;36(1):1–6. doi: 10.1203/00006450-199407001-00001 7936827

[20] Calderwood SK, Ciocca DR. Heat shock proteins: stress proteins with Janus-like properties in cancer. Int J Hyperthermia. 2008;24(1):31–9. doi: 10.1080/02656730701858305 18214767

[21] Jovaisaite V, Mouchiroud L, Auwerx J. The mitochondrial unfolded protein response, a conserved stress response pathway with implications in health and disease. J Exp Biol. 2014;217(1):137–43. 24353213 10.1242/jeb.090738 PMC3867496

[22] Hetz C. The unfolded protein response: controlling cell fate decisions under ER stress and beyond. Nat Rev Mol Cell Biol. 2012;13(2):89–102. doi: 10.1038/nrm3270 22251901

[23] Giglia-Mari G, Zotter A, Vermeulen W. DNA damage response. Cold Spring Harb Perspect Biol. 2011;3(1):a000745. doi: 10.1101/cshperspect.a000745 20980439 PMC3003462

[24] Hoeijmakers JH. Genome maintenance mechanisms for preventing cancer. Nature. 2001;411(6835):366–74. doi: 10.1038/35077232 11357144

[25] Hoeijmakers J. DNA damage, aging, and cancer. N Engl J Med. 2009;361(15):1475–85. 19812404 10.1056/NEJMra0804615

[26] Kulms D, Zeise E, Pöppelmann B, Schwarz T. DNA damage, death receptor activation and reactive oxygen species contribute to ultraviolet radiation-induced apoptosis in an essential and independent way. Oncogene. 2002;21(38):5844–51. doi: 10.1038/sj.onc.1205743 12185583

[27] Kulms D, Schwarz T. Molecular mechanisms of UV-induced apoptosis. Photodermatol Photoimmunol Photomed. 2000;16(5):195–201. 11068857 10.1034/j.1600-0781.2000.160501.x

[28] Timares L, Katiyar S, Elmets C. DNA damage, apoptosis and langerhans cells--activators of UV-induced immune tolerance. Photochem Photobiol. 2008;84(2):422–36. 18248501 10.1111/j.1751-1097.2007.00284.x PMC2718731

[29] Liang YG, Jorgensen AG, Kaestel CG, Wiencke AK, Lui GM, la Cour MH, et al. Bcl-2, Bax, and c-Fos expression correlates to RPE cell apoptosis induced by UV-light and daunorubicin. Curr Eye Res. 2000;20(1):25–34. doi: 10.1076/0271-3683(200001)2011-hft025 10611712

[30] Kannan K, Jain S. Oxidative stress and apoptosis. Pathophysiology. 2000;7(3):153–63. doi: 10.1016/s0928-4680(00)00053-5 10996508

[31] Gu ZT, Li L, Wu F, Zhao P, Yang H, Liu YS, et al. Heat stress induced apoptosis is triggered by transcription-independent p53, Ca(2+) dyshomeostasis and the subsequent Bax mitochondrial translocation. Sci Rep. 2015;5:11497. doi: 10.1038/srep11497 26105784 PMC4478470

[32] Ngo V, Duennwald ML. Nrf2 and oxidative stress: a general overview of mechanisms and implications in human disease. Antioxidants (Basel). 2022;11(12):2345. doi: 10.3390/antiox11122345 36552553 PMC9774434

[33] Itoh K, Chiba T, Takahashi S, Ishii T, Igarashi K, Katoh Y, et al. An Nrf2/small Maf heterodimer mediates the induction of phase II detoxifying enzyme genes through antioxidant response elements. Biochem Biophys Res Commun. 1997;236(2):313–22. doi: 10.1006/bbrc.1997.6943 9240432

[34] Moi P, Chan K, Asunis I, Cao A, Kan YW. Isolation of NF-E2-related factor 2 (Nrf2), a NF-E2-like basic leucine zipper transcriptional activator that binds to the tandem NF-E2/AP1 repeat of the beta-globin locus control region. Proc Natl Acad Sci U S A. 1994;91(21):9926–30. doi: 10.1073/pnas.91.21.9926 7937919 PMC44930

[35] Bowerman B, Eaton BA, Priess JR. skn-1, a maternally expressed gene required to specify the fate of ventral blastomeres in the early C. elegans embryo. Cell. 1992;68(6):1061–75. doi: 10.1016/0092-8674(92)90078-q 1547503

[36] Bowerman B, Draper BW, Mello CC, Priess JR. The maternal gene skn-1 encodes a protein that is distributed unequally in early C. elegans embryos. Cell. 1993;74(3):443–52. doi: 10.1016/0092-8674(93)80046-h 8348611

[37] An JH, Blackwell TK. SKN-1 links C. elegans mesendodermal specification to a conserved oxidative stress response. Genes Dev. 2003;17(15):1882–93. doi: 10.1101/gad.1107803 12869585 PMC196237

[38] Oliveira RP, Porter Abate J, Dilks K, Landis J, Ashraf J, Murphy CT, et al. Condition-adapted stress and longevity gene regulation by Caenorhabditis elegans SKN-1/Nrf. Aging Cell. 2009;8(5):524–41. doi: 10.1111/j.1474-9726.2009.00501.x 19575768 PMC2776707

[39] Pankiv S, Clausen TH, Lamark T, Brech A, Bruun J-A, Outzen H, et al. p62/SQSTM1 binds directly to Atg8/LC3 to facilitate degradation of ubiquitinated protein aggregates by autophagy. J Biol Chem. 2007;282(33):24131–45. doi: 10.1074/jbc.M702824200 17580304

[40] Ichimura Y, Kumanomidou T, Sou Y, Mizushima T, Ezaki J, Ueno T, et al. Structural basis for sorting mechanism of p62 in selective autophagy. J Biol Chem. 2008;283(33):22847–57. doi: 10.1074/jbc.M802182200 18524774

[41] Gatica D, Lahiri V, Klionsky DJ. Cargo recognition and degradation by selective autophagy. Nat Cell Biol. 2018;20(3):233–42. doi: 10.1038/s41556-018-0037-z 29476151 PMC6028034

[42] Baskoylu SN, Chapkis N, Unsal B, Lins J, Schuch K, Simon J, et al. Disrupted autophagy and neuronal dysfunction in C. elegans knockin models of FUS amyotrophic lateral sclerosis. Cell Rep. 2022;38(4):110195. 10.1016/j.celrep.2021.110195 35081350

[43] Jiang H, Ghose P, Han H, Wu Y, Tsai Y, Lin H. Blmp-1 promotes developmental cell death in C. elegans by timely repression of ced-9 transcription. Development. 2021;148(20). 10.1242/dev.193995 PMC8572009 34541605

[44] Shaham S, Reddien PW, Davies B, Horvitz HR. Mutational analysis of the Caenorhabditis elegans cell-death gene ced-3. Genetics. 1999;153(4):1655–71. doi: 10.1093/genetics/153.4.1655 10581274 PMC1460877

[45] Poteryaev D, Fares H, Bowerman B, Spang A. Caenorhabditis elegans SAND-1 is essential for RAB-7 function in endosomal traffic. EMBO J. 2007;26(2):301–12. doi: 10.1038/sj.emboj.7601498 17203072 PMC1783445

[46] Lee Y, Weihl CC. Regulation of SQSTM1/p62 via UBA domain ubiquitination and its role in disease. Autophagy. 2017;13(9):1615–6. doi: 10.1080/15548627.2017.1339845 28812433 PMC5612413

[47] Kulkarni M, Smith HE. E1 ubiquitin-activating enzyme UBA-1 plays multiple roles throughout C. elegans development. PLoS Genet. 2008;4(7):e1000131. doi: 10.1371/journal.pgen.1000131 18636104 PMC2443343

[48] Tian Y, Li Z, Hu W, Ren H, Tian E, Zhao Y, et al. C. elegans screen identifies autophagy genes specific to multicellular organisms. Cell. 2010;141(6):1042–55. doi: 10.1016/j.cell.2010.04.034 20550938

[49] Minnerly J, Zhang J, Parker T, Kaul T, Jia K. The cell non-autonomous function of ATG-18 is essential for neuroendocrine regulation of Caenorhabditis elegans lifespan. PLoS Genet. 2017;13(5):e1006764. doi: 10.1371/journal.pgen.1006764 28557996 PMC5469504

[50] Komatsu M, Kurokawa H, Waguri S, Taguchi K, Kobayashi A, Ichimura Y, et al. The selective autophagy substrate p62 activates the stress responsive transcription factor Nrf2 through inactivation of Keap1. Nat Cell Biol. 2010;12(3):213–23. doi: 10.1038/ncb2021 20173742

[51] Kobayashi A, Kang M-I, Okawa H, Ohtsuji M, Zenke Y, Chiba T, et al. Oxidative stress sensor Keap1 functions as an adaptor for Cul3-based E3 ligase to regulate proteasomal degradation of Nrf2. Mol Cell Biol. 2004;24(16):7130–9. doi: 10.1128/MCB.24.16.7130-7139.2004 15282312 PMC479737

[52] Cullinan SB, Gordan JD, Jin J, Harper JW, Diehl JA. The Keap1-BTB protein is an adaptor that bridges Nrf2 to a Cul3-based E3 ligase: oxidative stress sensing by a Cul3-Keap1 ligase. Mol Cell Biol. 2004;24(19):8477–86. 10.1128/MCB.24.19.8477-8486.2004 PMC516753 15367669

[53] Zhang DD, Lo S-C, Cross JV, Templeton DJ, Hannink M. Keap1 is a redox-regulated substrate adaptor protein for a Cul3-dependent ubiquitin ligase complex. Mol Cell Biol. 2004;24(24):10941–53. doi: 10.1128/MCB.24.24.10941-10953.2004 15572695 PMC533977

[54] McMahon M, Itoh K, Yamamoto M, Hayes JD. Keap1-dependent proteasomal degradation of transcription factor Nrf2 contributes to the negative regulation of antioxidant response element-driven gene expression. J Biol Chem. 2003;278(24):21592–600. doi: 10.1074/jbc.M300931200 12682069

[55] Itoh K, Wakabayashi N, Katoh Y, Ishii T, O’Connor T, Yamamoto M. Keap1 regulates both cytoplasmic-nuclear shuttling and degradation of Nrf2 in response to electrophiles. Genes Cells. 2003;8(4):379–91. doi: 10.1046/j.1365-2443.2003.00640.x 12653965

[56] Walker AK, See R, Batchelder C, Kophengnavong T, Gronniger JT, Shi Y, et al. A conserved transcription motif suggesting functional parallels between Caenorhabditis elegans SKN-1 and Cap’n’Collar-related basic leucine zipper proteins. J Biol Chem. 2000;275(29):22166–71. doi: 10.1074/jbc.M001746200 10764775

[57] Tang L, Dodd W, Choe K. Isolation of a Hypomorphic skn-1 Allele That Does Not Require a Balancer for Maintenance. G3 (Bethesda). 2015;6(3):551–8. doi: 10.1534/g3.115.023010 26715089 PMC4777118

[58] Lehrbach NJ, Breen PC, Ruvkun G. Protein Sequence Editing of SKN-1A/Nrf1 by Peptide:N-Glycanase Controls Proteasome Gene Expression. Cell. 2019;177(3):737-750.e15. doi: 10.1016/j.cell.2019.03.035 31002798 PMC6574124

[59] Lehrbach NJ, Ruvkun G. Proteasome dysfunction triggers activation of skn-1a/nrf1 by the aspartic protease ddi-1. Elife. 2016;5. 10.7554/eLife.17721 PMC4987142 27528192

[60] Nguyen CQ, Hall DH, Yang Y, Fitch DH. Morphogenesis of the Caenorhabditis elegans male tail tip. Dev Biol. 1999;207(1):86–106. 10049567 10.1006/dbio.1998.9173

[61] Crombie TA, Tang L, Choe KP, Julian D. Inhibition of the oxidative stress response by heat stress in Caenorhabditis elegans. J Exp Biol. 2016;219(Pt 14):2201–11. 27207646 10.1242/jeb.135327

[62] Przybysz AJ, Choe KP, Roberts LJ, Strange K. Increased age reduces DAF-16 and SKN-1 signaling and the hormetic response of Caenorhabditis elegans to the xenobiotic juglone. Mech Ageing Dev. 2009;130(6):357–69. doi: 10.1016/j.mad.2009.02.004 19428455 PMC2680786

[63] Senchuk MM, Dues DJ, Van Raamsdonk JM. Measuring Oxidative Stress in Caenorhabditis elegans: Paraquat and Juglone Sensitivity Assays. Bio Protoc. 2017;7(1):e2086. doi: 10.21769/BioProtoc.2086 29276721 PMC5739066

[64] Lo JY, Spatola BN, Curran SP. WDR23 regulates NRF2 independently of KEAP1. PLoS Genet. 2017;13(4):e1006762. doi: 10.1371/journal.pgen.1006762 28453520 PMC5428976

[65] Bishop NA, Guarente L. Two neurons mediate diet-restriction-induced longevity in C. elegans. Nature. 2007;447(7144):545–9. doi: 10.1038/nature05904 17538612

[66] Tang L, Choe KP. Characterization of skn-1/wdr-23 phenotypes in Caenorhabditis elegans; pleiotrophy, aging, glutathione, and interactions with other longevity pathways. Mech Ageing Dev. 2015;149:88–98. doi: 10.1016/j.mad.2015.06.001 26056713

[67] Barrett A, Hermann GJ. A Caenorhabditis elegans Homologue of LYST Functions in Endosome and Lysosome-Related Organelle Biogenesis. Traffic. 2016;17(5):515–35. doi: 10.1111/tra.12381 26822177

[68] Tchernev VT, Mansfield TA, Giot L, Kumar AM, Nandabalan K, Li Y, et al. The Chediak-Higashi protein interacts with SNARE complex and signal transduction proteins. Mol Med. 2002;8(1):56–64. doi: 10.1007/bf03402003 11984006 PMC2039936

[69] Han J, Pluhackova K, Bockmann R. The multifaceted role of SNARE proteins in membrane fusion. Front Physiol. 2017;8:5. 28163686 10.3389/fphys.2017.00005 PMC5247469

[70] Holland P, Torgersen ML, Sandvig K, Simonsen A. LYST affects lysosome size and quantity, but not trafficking or degradation through autophagy or endocytosis. Traffic. 2014;15(12):1390–405. doi: 10.1111/tra.12227 25216107

[71] Turner ME, Che J, Mirhaidari GJM, Kennedy CC, Blum KM, Rajesh S, et al. The lysosomal trafficking regulator “LYST”: an 80-year traffic jam. Front Immunol. 2024;15:1404846. doi: 10.3389/fimmu.2024.1404846 38774881 PMC11106369

[72] Thompson O, Edgley M, Strasbourger P, Flibotte S, Ewing B, Adair R, et al. The million mutation project: a new approach to genetics in Caenorhabditis elegans. Genome Res. 2013;23(10):1749–62. doi: 10.1101/gr.157651.113 23800452 PMC3787271

[73] Niu W, Lu ZJ, Zhong M, Sarov M, Murray JI, Brdlik CM, et al. Diverse transcription factor binding features revealed by genome-wide ChIP-seq in C. elegans. Genome Res. 2011;21(2):245–54. doi: 10.1101/gr.114587.110 21177963 PMC3032928

[74] Gerstein MB, Lu ZJ, Van Nostrand EL, Cheng C, Arshinoff BI, Liu T, et al. Integrative analysis of the Caenorhabditis elegans genome by the modENCODE project. Science. 2010;330(6012):1775–87. doi: 10.1126/science.1196914 21177976 PMC3142569

[75] Feng Y, Press B, Wandinger-Ness A. Rab 7: an important regulator of late endocytic membrane traffic. J Cell Biol. 1995;131(6 Pt 1):1435–52. doi: 10.1083/jcb.131.6.1435 8522602 PMC2120682

[76] Guo P, Hu T, Zhang J, Jiang S, Wang X. Sequential action of Caenorhabditis elegans Rab GTPases regulates phagolysosome formation during apoptotic cell degradation. Proc Natl Acad Sci U S A. 2010;107(42):18016–21. 20921409 10.1073/pnas.1008946107 PMC2964220

[77] Cantalupo G, Alifano P, Roberti V, Bruni CB, Bucci C. Rab-interacting lysosomal protein (RILP): the Rab7 effector required for transport to lysosomes. EMBO J. 2001;20(4):683–93. doi: 10.1093/emboj/20.4.683 11179213 PMC145419

[78] Poteryaev D, Datta S, Ackema K, Zerial M, Spang A. Identification of the switch in early-to-late endosome transition. Cell. 2010;141(3):497–508. doi: 10.1016/j.cell.2010.03.011 20434987

[79] Guo P, Wang X. Rab GTPases act in sequential steps to regulate phagolysosome formation. Small GTPases. 2010;1(3):170–3. doi: 10.4161/sgtp.1.3.14511 21686272 PMC3116604

[80] Kundra R, Kornfeld S. Asparagine-linked oligosaccharides protect Lamp-1 and Lamp-2 from intracellular proteolysis. J Biol Chem. 1999;274(43):31039–46. 10521503 10.1074/jbc.274.43.31039

[81] Kannan K, Stewart RM, Bounds W, Carlsson SR, Fukuda M, Betzing KW, et al. Lysosome-associated membrane proteins h-LAMP1 (CD107a) and h-LAMP2 (CD107b) are activation-dependent cell surface glycoproteins in human peripheral blood mononuclear cells which mediate cell adhesion to vascular endothelium. Cell Immunol. 1996;171(1):10–9. doi: 10.1006/cimm.1996.0167 8660832

[82] Saftig P, Klumperman J. Lysosome biogenesis and lysosomal membrane proteins: trafficking meets function. Nat Rev Mol Cell Biol. 2009;10(9):623–35. doi: 10.1038/nrm2745 19672277

[83] Kostich M, Fire A, Fambrough DM. Identification and molecular-genetic characterization of a LAMP/CD68-like protein from Caenorhabditis elegans. J Cell Sci. 2000;113 ( Pt 14):2595–606. doi: 10.1242/jcs.113.14.2595 10862717

[84] Sala AJ, Bott LC, Brielmann RM, Morimoto RI. Embryo integrity regulates maternal proteostasis and stress resilience. Genes Dev. 2020;34(9–10):678–87. doi: 10.1101/gad.335422.119 32217667 PMC7197353

[85] Ewe CK, Alok G, Rothman JH. Stressful development: integrating endoderm development, stress, and longevity. Dev Biol. 2021;471:34–48. doi: 10.1016/j.ydbio.2020.12.002 33307045

[86] Byun YG, Kim N-S, Kim G, Jeon Y-S, Choi JB, Park C-W, et al. Stress induces behavioral abnormalities by increasing expression of phagocytic receptor MERTK in astrocytes to promote synapse phagocytosis. Immunity. 2023;56(9):2105-2120.e13. doi: 10.1016/j.immuni.2023.07.005 37527657

[87] Anderson HA, Englert R, Gursel I, Shacter E. Oxidative stress inhibits the phagocytosis of apoptotic cells that have externalized phosphatidylserine. Cell Death Differ. 2002;9(6):616–25. doi: 10.1038/sj.cdd.4401013 12032670

[88] Morrish F, Giedt C, Hockenbery D. c-MYC apoptotic function is mediated by NRF-1 target genes. Genes Dev. 2003;17(2):240–55. doi: 10.1101/gad.1032503 12533512 PMC195978

[89] Choi HI, Kim HJ, Park JS, Kim IJ, Bae EH, Ma SK, et al. Pgc-1alpha attenuates hydrogen peroxide-induced apoptotic cell death by upregulating nrf-2 via gsk3beta inactivation mediated by activated p38 in hk-2 cells. Sci Rep. 2017;7(1):4319. 28659586 10.1038/s41598-017-04593-w PMC5489530

[90] Wang Y, Tang B, Li H, Zheng J, Zhang C, Yang Z, et al. A small-molecule inhibitor of Keap1-Nrf2 interaction attenuates sepsis by selectively augmenting the antibacterial defence of macrophages at infection sites. EBioMedicine. 2023;90:104480. doi: 10.1016/j.ebiom.2023.104480 36863256 PMC9996215

[91] Gulyas L, Powell JR. Cold shock induces a terminal investment reproductive response in C. elegans. Sci Rep. 2022;12(1):1338. doi: 10.1038/s41598-022-05340-6 35079060 PMC8789813

[92] Tarpey PS, Behjati S, Young MD, Martincorena I, Alexandrov LB, Farndon SJ, et al. The driver landscape of sporadic chordoma. Nat Commun. 2017;8(1):890. doi: 10.1038/s41467-017-01026-0 29026114 PMC5638846

[93] Bong IPN, Ng CC, Fakiruddin SK, Lim MN, Zakaria Z. Small interfering RNA-mediated silencing of nicotinamide phosphoribosyltransferase (NAMPT) and lysosomal trafficking regulator (LYST) induce growth inhibition and apoptosis in human multiple myeloma cells: A preliminary study. Bosn J Basic Med Sci. 2016;16(4):268–75. doi: 10.17305/bjbms.2016.1568 27754828 PMC5136762

[94] Zhao Q, Wang F, Chen Y-X, Chen S, Yao Y-C, Zeng Z-L, et al. Comprehensive profiling of 1015 patients’ exomes reveals genomic-clinical associations in colorectal cancer. Nat Commun. 2022;13(1):2342. doi: 10.1038/s41467-022-30062-8 35487942 PMC9055073

[95] Li L, Zheng H, Huang Y, Huang C, Zhang S, Tian J, et al. DNA methylation signatures and coagulation factors in the peripheral blood leucocytes of epithelial ovarian cancer. Carcinogenesis. 2017;38(8):797–805. doi: 10.1093/carcin/bgx057 28637314 PMC7083145

[96] Huang Z, Zhang H, Xing C, Zhang L, Zhu H, Deng Z, et al. Identification and validation of CALCRL-associated prognostic genes in acute myeloid leukemia. Gene. 2022;809:146009. doi: 10.1016/j.gene.2021.146009 34655717

[97] Li F, Hu S, Kong K, Cao P, Han P, Deng Y, et al. Next-Generation Sequencing Analysis Identified Genomic Alterations in Pathological Morphologies of 3 Cases of Pulmonary Carcinosarcoma. Onco Targets Ther. 2020;13:7963–72. doi: 10.2147/OTT.S264617 32848420 PMC7429410

[98] Ivyna Bong PN, Ng CC, Lam KY, Megat Baharuddin PJN, Chang KM, Zakaria Z. Identification of novel pathogenic copy number aberrations in multiple myeloma: the Malaysian context. Mol Cytogenet. 2014;7(1):24. doi: 10.1186/1755-8166-7-24 24690091 PMC4021726

[99] Zbinden JC, Mirhaidari GJM, Blum KM, Musgrave AJ, Reinhardt JW, Breuer CK, et al. The lysosomal trafficking regulator is necessary for normal wound healing. Wound Repair Regen. 2022;30(1):82–99. doi: 10.1111/wrr.12984 34837653 PMC9004365

[100] Fukai K, Oh J, Karim MA, Moore KJ, Kandil HH, Ito H, et al. Homozygosity mapping of the gene for Chediak-Higashi syndrome to chromosome 1q42-q44 in a segment of conserved synteny that includes the mouse beige locus (bg). Am J Hum Genet. 1996;59(3):620–4. 8751863 PMC1914913

[101] Barrat FJ, Auloge L, Pastural E, Lagelouse RD, Vilmer E, Cant AJ. Genetic and physical mapping of the Chediak-Higashi syndrome on chromosome 1q42-43. Am J Hum Genet. 1996;59(3):625–32. 8751864 PMC1914920

[102] Ward DM, Shiflett SL, Kaplan J. Chediak-Higashi syndrome: a clinical and molecular view of a rare lysosomal storage disorder. Curr Mol Med. 2002;2(5):469–77. doi: 10.2174/1566524023362339 12125812

[103] Perou C, Moore K, Nagle D, Misumi D, Woolf E, McGrail S. Identification of the murine beige gene by YAC complementation and positional cloning. Nat Genet. 1996;13(3):303–8. 8673129 10.1038/ng0796-303

[104] Mahoney KH, Morse SS, Morahan PS. Macrophage functions in beige (Chédiak-Higashi syndrome) mice. Cancer Res. 1980;40(11):3934–9. 7471044

[105] Westphal A, Cheng W, Yu J, Grassl G, Krautkramer M, Holst O. Lysosomal trafficking regulator lyst links membrane trafficking to toll-like receptor-mediated inflammatory responses. J Exp Med. 2017;214(1):227–44. 27881733 10.1084/jem.20141461 PMC5206490

[106] Choe KP, Przybysz AJ, Strange K. The WD40 repeat protein WDR-23 functions with the CUL4/DDB1 ubiquitin ligase to regulate nuclear abundance and activity of SKN-1 in Caenorhabditis elegans. Mol Cell Biol. 2009;29(10):2704–15. doi: 10.1128/MCB.01811-08 19273594 PMC2682033

[107] Liu J, Duangjan C, Irwin RW, Curran SP. WDR23 mediates NRF2 proteostasis and cytoprotective capacity in the hippocampus. Mech Ageing Dev. 2024;218:111914. doi: 10.1016/j.mad.2024.111914 38301772 PMC10939789

[108] Kansanen E, Kuosmanen SM, Leinonen H, Levonen A-L. The Keap1-Nrf2 pathway: Mechanisms of activation and dysregulation in cancer. Redox Biol. 2013;1(1):45–9. doi: 10.1016/j.redox.2012.10.001 24024136 PMC3757665

[109] Arnold M, Cooper J, Androwski R, Ardeshna S, Melentijevic I, Smart J. Intermediate filaments associate with aggresome-like structures in proteostressed C. elegans neurons and influence large vesicle extrusions as exophers. Nat Commun. 2023;14(1):4450. 37488107 10.1038/s41467-023-39700-1 PMC10366101

[110] Dokshin GA, Ghanta KS, Piscopo KM, Mello CC. Robust Genome Editing with Short Single-Stranded and Long, Partially Single-Stranded DNA Donors in Caenorhabditis elegans. Genetics. 2018;210(3):781–7. doi: 10.1534/genetics.118.301532 30213854 PMC6218216

