# Supplementary figures and images for: SQST-1/p62-regulated SKN-1/Nrf mediates a phagocytic stress response via transcriptional activation of lyst-1/LYST
Source: PLoS Genet. 2025 May 2;21(5):e1011696. doi: 10.1371/journal.pgen.1011696 (PMC12068719; doi:10.1371/journal.pgen.1011696)

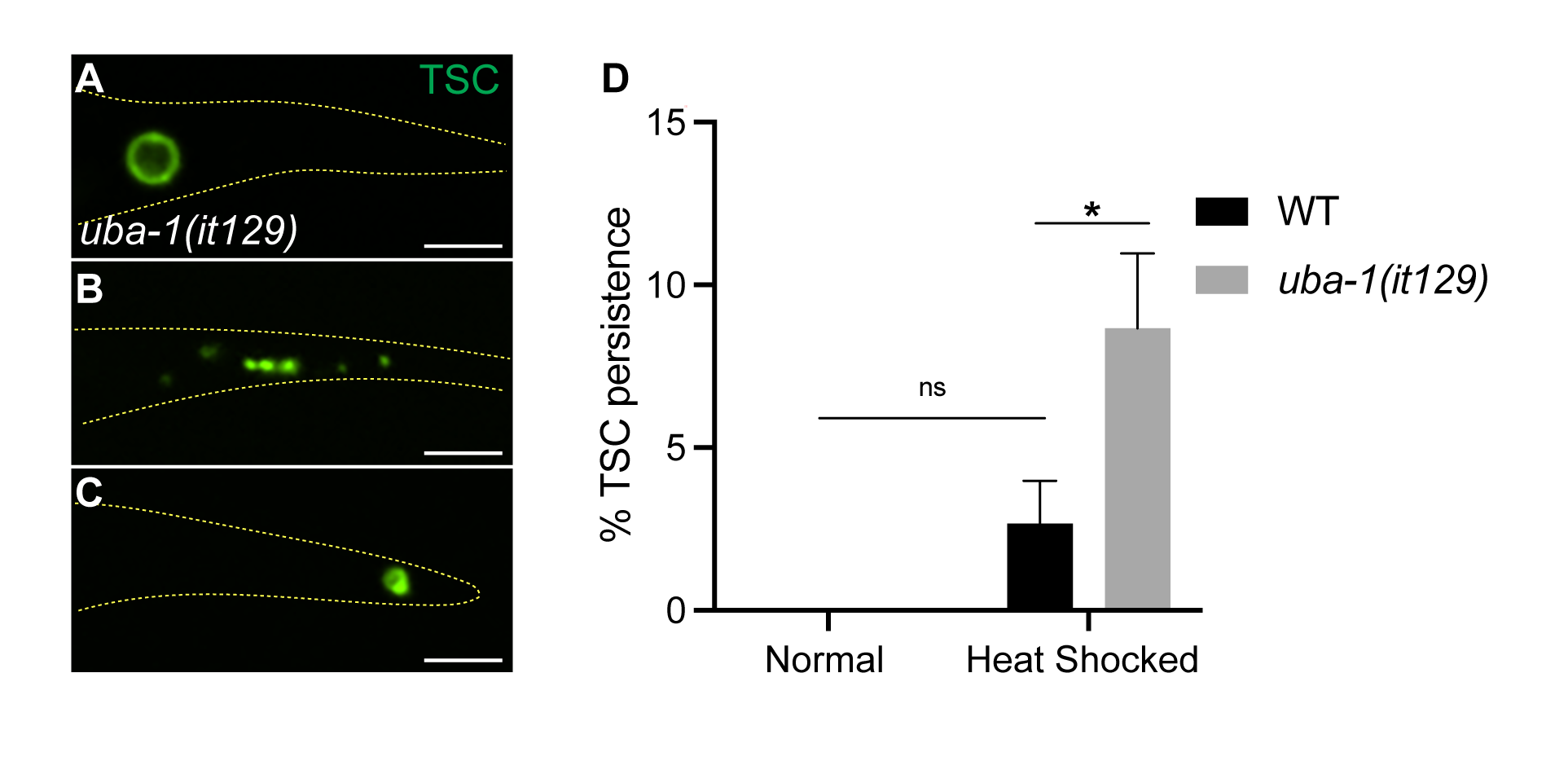

Supplement: S1 Fig — (A-C) uba-1(it129) mutant CCE defects following heat stress. (D) Quantification of TSC persistence in wild-type vs uba-1(it129). N > 50. ns (not significant) p > 0.05, * p ≤ 0.05, ** p ≤ 0.01, *** p ≤ 0.001, **** p ≤ 0.0001. (TIF) [file pgen.1011696.s001.tif]

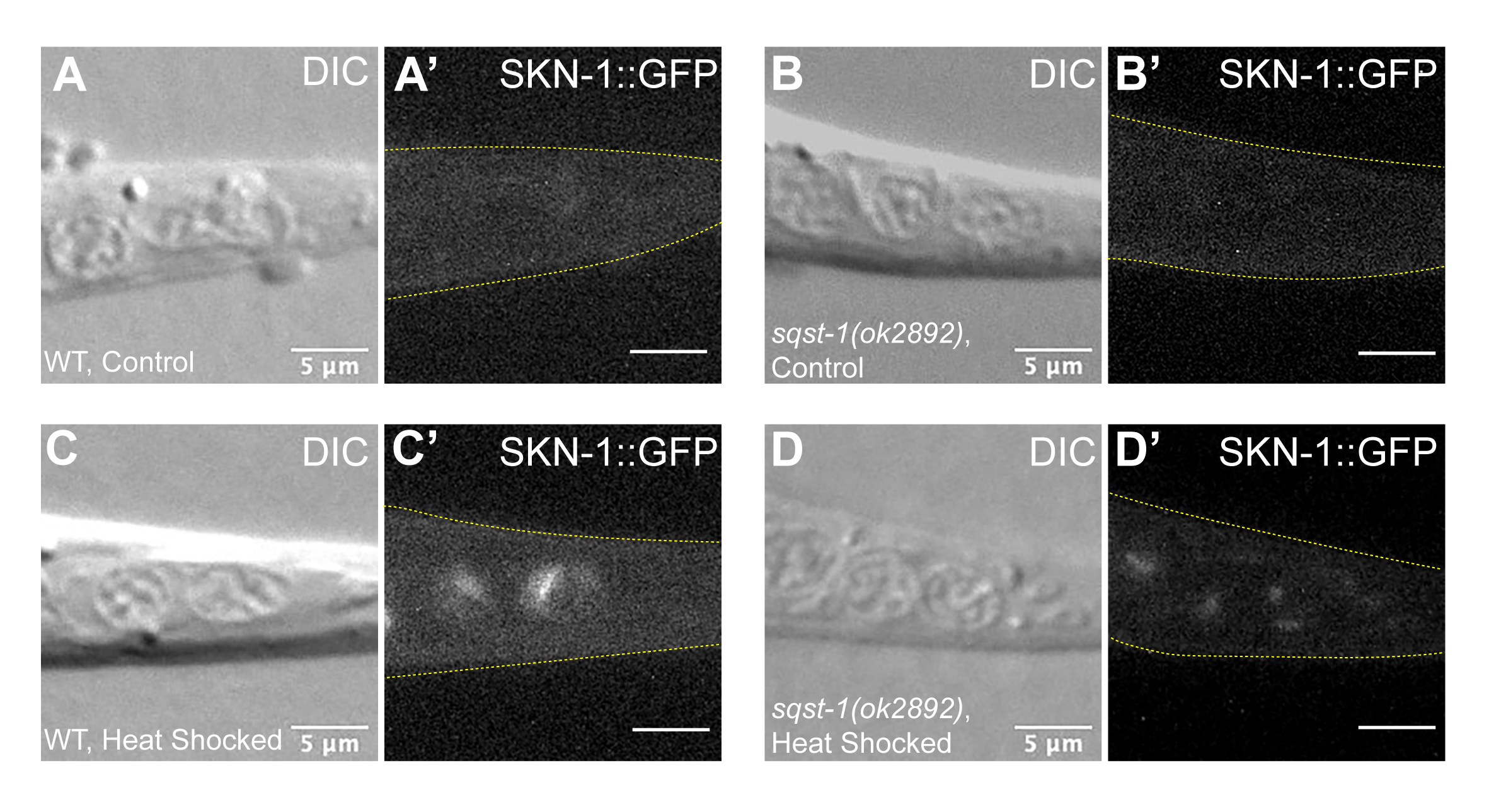

Supplement: S2 Fig — N = 10 for all. (TIF) [file pgen.1011696.s002.tif]

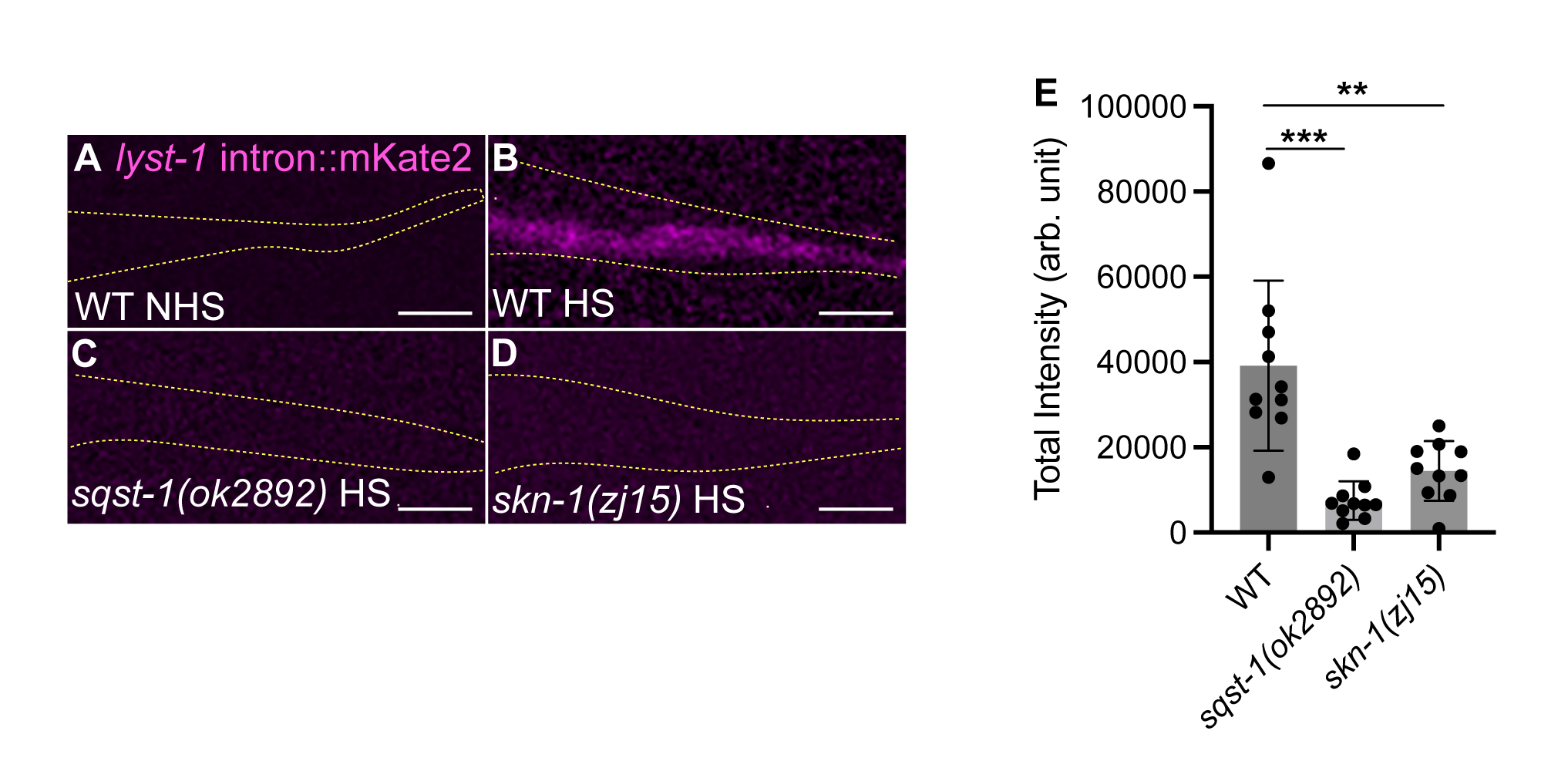

Supplement: S3 Fig — (A) Wild-type animal showing no expression of lyst-1/LYST in hyp10 under basal conditions. N = 10. (B) Wild-type animal showing expression of lyst-1/LYST in hyp10 following heat stress. N = 10. (C) sqst-1(ok2892) mutant showing no expression of lyst-1/LYST in hyp10 following heat stress. N = 10. (D) skn-1(zj15) mutant showing no expression of lyst-1/LYST in hyp10 following heat stress. N = 10. (E) Quantification of B-D. ns (not significant) p > 0.05, * p ≤ 0.05, ** p ≤ 0.01, *** p ≤ 0.001, **** p ≤ 0.0001. (TIF) [file pgen.1011696.s003.tif]

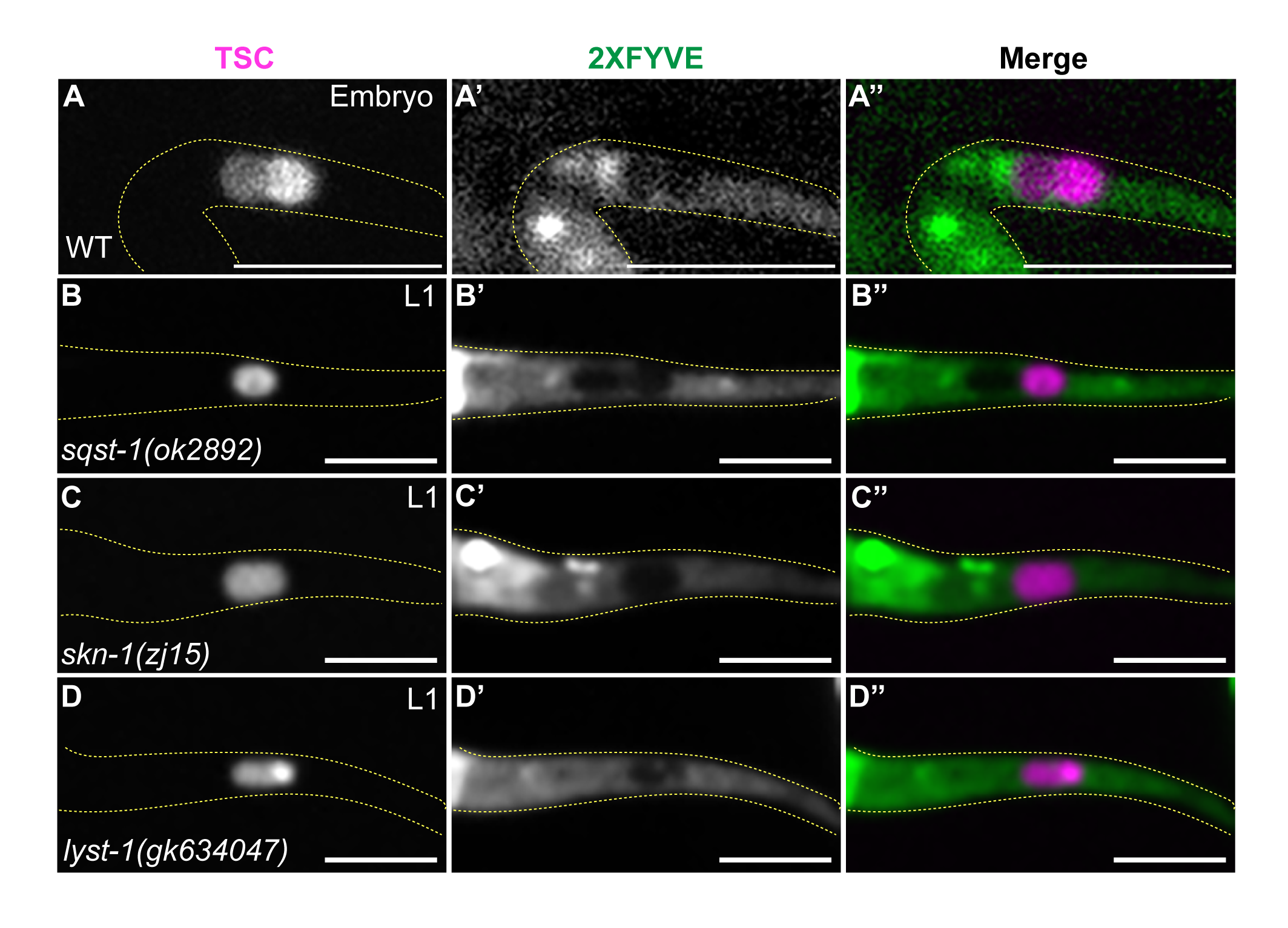

Supplement: S4 Fig — (TIF) [file pgen.1011696.s004.tif]

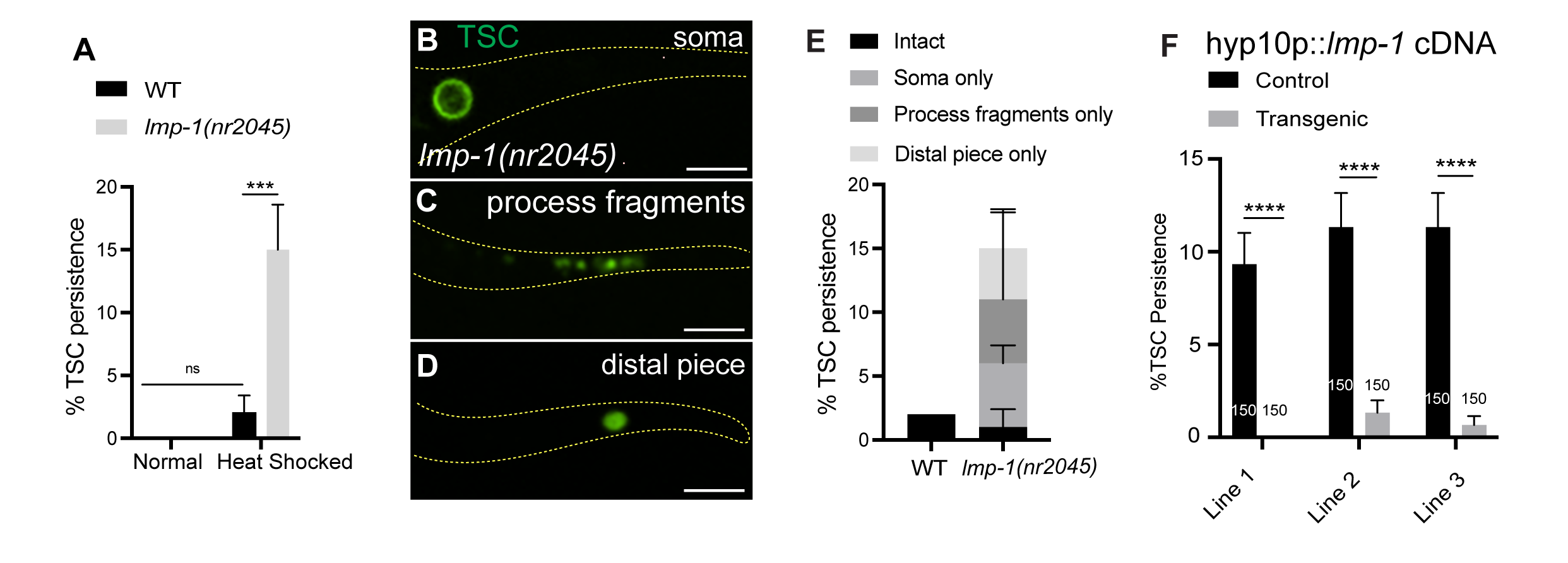

Supplement: S5 Fig — N > 50. (B-D) lmp-1(nr2045) mutant CCE defects following heat stress. (E) Quantification of lmp-1(-) phenotype categories. (F) hyp10-specific rescue of lmp-1(nr2045) defect. ns (not significant) p > 0.05, * p ≤ 0.05, ** p ≤ 0.01, *** p ≤ 0.001, **** p ≤ 0.0001. (TIF) [file pgen.1011696.s005.tif]
