# Supplementary material for: SQST-1/p62-regulated SKN-1/Nrf mediates a phagocytic stress response via transcriptional activation of lyst-1/LYST
Source: PLoS Genet. 2025 May 2;21(5):e1011696. doi: 10.1371/journal.pgen.1011696 (PMC12068719; doi:10.1371/journal.pgen.1011696)
Supplement: S1 Table — (PDF) [file pgen.1011696.s007.pdf]

Supplement Table 1: Plasmids used in this study

Plasmid number	Primers used	Sequences
pPG302 (*sqst-1* expression)	oAE15 mKate2 Vector backbone_F	ATGGTCTCCGAGCTCATTAAcGAAAAC
	oAE16 mKate2 Vector backbone_R	GATCCTCTAGAGTCGACCTGCAGGC
	oAE17 *sqst-1* pro insert-F	GAAATAAGCTTGCATGCCTGCAGGTCGACTCTAGAG GATCagatcattcaaagatgaaatccattagatt
	oAE18 *sqst-1* pro insert-R	GCTTCATATGCATGTTTTCgTTAATGAGCTCGGAGAC CATctgagtaaaatgagaagttatttgaaagtg
pPG312 (*skn-1* expression)	oAE15 mKate2 Vector backbone_F	ATGGTCTCCGAGCTCATTAAcGAAAAC
	oAE16 mKate2 Vector backbone_R	GATCCTCTAGAGTCGACCTGCAGGC
	oAE31 *skn-1* pro insert_F	GAAATAAGCTTGCATGCCTGCAGGTCGACTCTAGAG GATCtgctcacagatctcaaagctgcgtgtg
	oAE32 *skn-1* pro insert_R	GCTTCATATGCATGTTTTCgTTAATGAGCTCGGAGAC CATctgaaaatttggaattatttttgggaatatcg
pPG313 (*sqst-1* tsc rescue)	oYM14 *sqst-1* gDNA insert_F	tccatactttctcatttcataatattcggaacGGCGCGCCaaaATGGC TGCTGCATCATCCGCTCC
	oYM15 *sqst-1* gDNA insert_R	tacctttgggtcctttggccaatcccggggatcctctagaTTAGTGAAG AAGCGCCTGAAGACACATC
	oKJ43 TSCpro backbone_F	GGCGCGCCgttccgaatattatg
	oKJ44 TSCpro backbone_R	GAATTCcaactgagcgccggtc
pPG325 (*skn-1* tsc rescue)	oYM37 TSCpro backbone_F	tctagaggatccccgggattggcc

	oYM38 TSCpro backbone_R	GGCGCGCCgttccgaatattatgaaa
	oYM39 *skn-1a* cDNA insert_F	tccatactttctcatttcataatattcggaacGGCGCGCCaaaATGGGCGGTTCATCACGCCGTCA
	oYM40 *skn-1a* cDNA insert_R	tacctttgggtcctttggccaatcccggggatcctctagaTCAGATGTAATGGGACATCTTGTCGTG
pPG336 (*lyst-1* intron expression)	oAE40 mKate2 Vector backbone_F	ATGGTCTCCGAGCTCATTAAcGAAAAC
	oAE41 mKate2 Vector backbone_R	GATCCTCTAGAGTCGACCTGCAGGCATG
	oAE44 *lyst-1* intron insert_F	GAAATAAGCTTGCATGCCTGCAGGTCGACTCTAGAGGATCtgctttttatatttcgttttaatgttttgcag
	oAE45 *lyst-1* intron insert_R	GCTTCATATGCATGTTTTCgTTAATGAGCTCGGAGACCATctgaaaaaataataactttttaaaccctaac
pPG359 (*sqst-1* hyp10 rescue)	oAE70 vector backbone_F	GGCCGGCCcagtcagtgcggccgc
	oAE71 vector backbone_R	gctgtctcatcctactttcacctagttaac
	oAE72 hyp10pro insert_F	cgccaagcttgcatgcgcggccgcactgactgGGCCGGCCttttgatactttttaatcaaaaagtttaccgc
	oAE73 hyp10pro insert_R	GAACTTGCATTTGGTGAGGAGCGGATGATGCAGCAGCCATttttaaaacaaaaaaagatgccttcctattg
	oAE74 *sqst-1* gDNA insert_F	gttagtttccaataggaaggcatctttttttgttttaaaaATGGCTGCTGCATCATCCGCTCCTCAC
	oAE75 *sqst-1* gDNA insert_R	aagacaagcagttaactaggtgaaagtaggatgagacagcTTAGTGAAGAAGCGCCTGAAGACACATC
pPG360 (*skn-1* hyp10 rescue)	oAE70 vector backbone_F	GGCCGGCCcagtcagtgcggccgc

	oAE71 vector backbone_R	gctgtctcatcctactttcacctagttaac
	oAE76 hyp10pro backbone_F	cgccaagcttgcatgcgcggccgcactgactgGGCCGGCCttttgatactttttaatcaaaaagtttaccgc
	oAE77 hyp10pro backbone_R	CCGACGTACTTCGCTGACGGCGTGATGAACCGCCCATttttttaaaacaaaaaaagatgccttcctattgg
	oAE78 *skn-1a* cDNA insert_F	gttagtttccaataggaaggcatctttttttgttttaaaaaaATGGGCGGTTCATCACGCCGTCAGC
	oAE79 *skn-1a* cDNA insert_R	aagacaagcagttaactaggtgaaagtaggatgagacagcTCAGATGTAATGGGACATCTTGTCGTGAC
pPG370b (*lyst-1* hyp10 rescue)	oAE98 hyp10pro backbone_F	gctgtctcatcctactttcacctagtt
	oAE99 hyp10pro backbone_R	ttttaaaacaaaaaaagatgccttcctattg
	oAE114 *lyst-1* cDNA (1st half) insert_F	gttagtttccaataggaaggcatctttttttgttttaaaaaaATGGAAAAGATCCGCTCACCATCACT
	oAE115 *lyst-1* cDNA (1st half) insert_R	TAGATTTAATTGGACGATTTTTGTTATCTTGTCCGAGTAAATGTTATGGTTAATAATAGCTTCATACATCTC
	oAE116 *lyst-1* cDNA (2nd half) insert_F	CCCTCATAGAGATGTATGAAGCTATTATTAACCATAACATTTACTCGGACAAGATAACAAAAATCGTCC
	oAE117 *lyst-1* cDNA (2nd half) insert_R	aagacaagcagttaactaggtgaaagtaggatgagacagcTCAAGTTCTTATTTTGAATCTCCACGTTTTG
pPG404 (*lmp-1* hyp10 rescue)	oAE138 hyp10pro backbone_F	gctgtctcatcctactttcacctagtt
	oAE139 hyp10pro backbone_R	ttttaaaacaaaaaaagatgccttcctattg

	oAE140 *lmp-1* cDNA insert_F	gttagtttccaataggaaggcatctttttttgttttaaaaaaaATGTTGAAATCGTTTGTCATCTTGTTTG
	oAE141 *lmp-1* cDNA insert_R	aagacaagcagttaactaggtgaaagtaggatgagacagcTTAGACGCTGGCATATCCTTGTCTC
pPG422 (*wdr-23* Overexpression)	oAE163 hyp10pro backbone_F	ttttaaaacaaaaaaagatgccttcctattgg
	oAE164 hyp10pro backbone_R	gctgtctcatcctactttcacctagttaac
	oAE165 *wdr-23* cDNA insert_F	gttagtttccaataggaaggcatctttttttgttttaaaaaaaATGGGCAACTGGATAACGTCGACG
	oAE166 *wdr-23* cDNA insert_R	aagacaagcagttaactaggtgaaagtaggatgagacagcTTAATTTTGAGAGATGCTGCTCGATGAGC
