# Supplementary material for: SQST-1/p62-regulated SKN-1/Nrf mediates a phagocytic stress response via transcriptional activation of lyst-1/LYST
Source: PLoS Genet. 2025 May 2;21(5):e1011696. doi: 10.1371/journal.pgen.1011696 (PMC12068719; doi:10.1371/journal.pgen.1011696)
Supplement: S2 Table — (PDF) [file pgen.1011696.s008.pdf]

Supplement Table 2: List of transgenes and strains

Strain	Genotype	Comments
TSC125	T12G3.1(ok2869); *nsIs435*	T12G3.1=*sqst-1; nsIs435=aff-1*p::myrGFP
TSC126	T12G3.1(ok2892); *nsIs435*	T12G3.1=*sqst-1; nsIs435=aff-1*p::myrGFP
TSC148	*skn-1(zj15); nsIs435*	*nsIs435=aff-1*p::myrGFP
TSC149	*skn-1(mg570); nsIs435*	*nsIs435=aff-1*p::myrGFP
TSC156	*uba-1(it129); nsIs435*	*nsIs435=aff-1*p::myrGFP
TSC174	*ced-3(n717); nsIs435; mccEx096*	*mccEx096*=pPG312; *nsIs435=aff-1*p::myrGFP
TSC175	*sqst-1(ok2892); nsIs435; mccEx097*	*mccEx097*=pPG313; *nsIs435=aff-1*p::myrGFP
TSC176	*sqst-1(ok2892); nsIs435; mccEx098*	*mccEx098*=pPG313; *nsIs435=aff-1*p::myrGFP
TSC177	*sqst-1(ok2892); nsIs435; mccEx099*	*mccEx099*=pPG313; *nsIs435=aff-1*p::myrGFP
TSC193	*ced-3(n717); nsIs435; mccEx110*	*mccEx110*=pPG302; *nsIs435=aff-1*p::myrGFP
TSC214	*lyst-1(gk803491); nsIs435*	*nsIs435=aff-1*p::myrGFP
TSC215	*lyst-1(gk634047); nsIs435*	*nsIs435=aff-1*p::myrGFP
TSC220	*ced-3(n717); nsIs435; mccEx126*	*mccEx126*=pPG336; *nsIs435=aff-1*p::myrGFP
TSC221	*ced-3(n717); nsIs435; mccEx127*	*mccEx127*=pPG336; *nsIs435=aff-1*p::myrGFP
TSC222	*ced-3(n717); nsIs435; mccEx128*	*mccEx128*=pPG336; *nsIs435=aff-1*p::myrGFP
TSC223	*skn-1(zj15); nsIs435; mccEx119*	*mccEx119*=pPG325; *nsIs435=aff-1*p::myrGFP
TSC224	*skn-1(zj15); nsIs435; mccEx120*	*mccEx120*=pPG325; *nsIs435=aff-1*p::myrGFP
TSC225	*skn-1(zj15); nsIs435; mccEx121*	*mccEx121*=pPG325; *nsIs435=aff-1*p::myrGFP
TSC232	*lmp-1(nr2045); nsIs435*	*nsIs435=aff-1*p::myrGFP
TSC254	*ced-3(n717); mccIs017*	*mccIs017*=pPG336
TSC265	*sqst-1(mcc13); nsIs435*	*mcc13=sqst-1(mcc13)*.1358 G -> A; *nsIs435=aff-1*p::myrGFP.
TSC279	T12G3.1(ok2892); *lyst-1(gk634047); nsIs435*	T12G3.1=*sqst-1*; *nsIs435=aff-1*p::myrGFP
TSC280	*skn-1(zj15); lyst-1(gk634047); nsIs435*	*nsIs435=aff-1*p::myrGFP
TSC281	N2; *mccIs017; nsIs435*	*mccIs017*=pPG336
TSC283	*atg-13(bp414); nsIs435*	*nsIs435=aff-1*p::myrGFP
TSC284	*atg-18(gk378);nsIs431*	F41E6.13a. Superficially wild type. *nsIs431=aff-1*p::myrGFP
TSC315	T12G3.1(ok2892); *nsIs435; mccEx161*	*mccEx161*=pPG359; T12G3.1=*sqst-1*; *nsIs435=aff-1*p::myrGFP

TSC316	T12G3.1(ok2892); *nsIs435; mccEx162*	*mccEx162*=pPG359; T12G3.1=*sqst-1*; *nsIs435=aff-1*p::myrGFP
TSC317	T12G3.1(ok2892); *nsIs435; mccEx163*	*mccEx163*=pPG359; T12G3.1=*sqst-1*; *nsIs435=aff-1*p::myrGFP
TSC318	*skn-1(zj15); nsIs435; mccEx164*	*mccEx164*=pPG360; *nsIs435=aff-1*p::myrGFP
TSC319	*skn-1(zj15); nsIs435; mccEx165*	*mccEx165*=pPG360; *nsIs435=aff-1*p::myrGFP
TSC320	*skn-1(zj15); nsIs435; mccEx166*	*mccEx166*=pPG360; nsIs435=*aff-1*p::myrGFP
TSC324	T12G3.1(ok2892); mccIs017; nsIs435	*mccIs017*=pPG336; T12G3.1=*sqst-1*; *nsIs435=aff-1*p::myrGFP
TSC370	*skn-1(zj15); mccIs017; nsIs435*	*mccIs017*=pPG336; *nsIs435=aff-1*p::myrGFP
TSC392	*lyst-1(gk634047); nsIs435; mccEx210*	*mccEx210*=pPG370b; *nsIs435=aff-1*p::myrGFP
TSC393	*lyst-1(gk634047); nsIs435; mccEx211*	*mccEx211*=pPG370b; *nsIs435=aff-1*p::myrGFP
TSC394	*lyst-1(gk634047); nsIs435; mccEx212*	*mccEx212*=pPG370b; *nsIs435=aff-1*p::myrGFP
TSC429	*nsIs685; nsIs836*	*nsIs685=aff-1*p::mKate2; *nsIs836=eff-1*p::iBlueberry
TSC430	T12G3.1(ok2892); *nsIs685; nsIs836*	T12G3.1=*sqst-1*; *nsIs685=aff-1*p::mKate2; *nsIs836=eff-1*p::iBlueberry
TSC432	*skn-1(zj15); nsIs685; nsIs836*	*nsIs685=aff-1*p::mKate2; *nsIs836=eff-1*p::iBlueberry
TSC438	N2;*nsIs435;nsEx5971*	*nsIs435=aff-1*p::myrGFP; *nsEx5971=ced-1*p::LAAT-1::mCherry
TSC439	N2;*nsIs685;nsEx5975*	*nsIs685=aff-1*p::mKate2; *nsEx5975=eff-1*p::GFP::RAB-7
TSC473	*lmp-1(nr2045); nsIs435; mccEx234*	*mccEx234*=pPG404
TSC474	*lmp-1(nr2045); nsIs435; mccEx235*	*mccEx235*=pPG404
TSC475	*lmp-1(nr2045); nsIs435; mccEx236*	*mccEx236*=pPG404
TSC480	T12G3.1(ok2892); *nsIs435; nsEx5971*	T12G3.1=*sqst-1*; *nsEx5971=ced-1*p::LAAT-1::mCherry; *nsIs435=aff-1*p::myrGFP
TSC481	*skn-1(zj15); nsIs685; nsEx5975*	*nsEx5975=eff-1*p::GFP::RAB-7; *nsIs685=aff-1*p::mKate2
TSC511	*skn-1(zj15); nsIs435; mccEx241*	*mccEx241*=pPG370b; *nsIs435=aff-1*p::myrGFP
TSC512	*skn-1(zj15); nsIs435; mccEx242*	*mccEx242*=pPG370b; *nsIs435=aff-1*p::myrGFP
TSC513	*skn-1(zj15); nsIs435; mccEx243*	*mccEx243*=pPG370b; *nsIs435=aff-1*p::myrGFP

TSC585	*nsIs435; mccEx247*	*mccEx247*=pPG422; *nsIs435=aff-1*p::myrGFP
TSC586	*skn-1(zu169); geIs7; nsIs685; nsIs836*	*geIs7* [*skn-1b*::GFP]; *nsIs685=aff-1*p::mKate2; *nsIs836=eff-1*p::iBlueberry
TSC587	T12G3.1(ok2892); *geIs7; nsIs685; nsIs836*	T12G3.1=*sqst-1*; *geIs7* [*skn-1b*::GFP]; *nsIs685=aff-1*p::mKate2; *nsIs836=eff-1*p::iBlueberry
TSC589	T12G3.1(ok2892); *nsIs685; nsEx5975*	T12G3.1=*sqst-1*; *nsEx5975=eff-1*p::GFP::RAB-7; *nsIs685=aff-1*p::mKate2
TSC590	*skn-1(zj15); nsIs435; nsEx5971*	*nsEx5971=ced-1*p::LAAT-1::mCherry; *nsIs435=aff-1*p::myrGFP
TSC591	*lyst-1(gk634047); nsIs435; nsEx5971*	*nsEx5971=ced-1*p::LAAT-1::mCherry; *nsIs435=aff-1*p::myrGFP
TSC625	*nsIs435; mccEx256*	*mccEx256*=pPG422; *nsIs435=aff-1*p::myrGFP
TSC626	*nsIs435; mccEx257*	*mccEx257*=pPG422; *nsIs435=aff-1*p::myrGFP
TSC656	*lyst-1(gk634047); mccIs099; nsEx5975*	*mccIs099=aff-1*p::myrmCherry; *nsEx5975=eff-1*p::GFP::RAB-7
TSC669	*lyst-1(gk634047); mccIs099; mccEx062*	*mccIs099=aff-1*p::myrmCherry; *mccEx062=eff-1*p(-4200 ->-4450)::GFP
TSC719	*wdr-23(mcc37)*	*mcc37*=WDR-23::GFP, insertion at N term.
TSC720	T12G3.1(ok2892); *wdr-23(mcc37)*	T12G3.1=*sqst-1; mcc37*=WDR-23::GFP, insertion at N term.
TSC721	*nsIs685; mccEx281*	*nsIs685=aff-1*p::mKate2; *mccEx281*=2XFYVE::GFP
TSC722	T12G3.1(ok2892); *nsIs685; mccEx281*	T12G3.1=*sqst-1; nsIs685=aff-1*p::mKate2; *mccEx281*=2XFYVE::GFP
TSC723	*skn-1(zj15); nsIs685; mccEx281*	*nsIs685=aff-1*p::mKate2; *mccEx281*=2XFYVE::GFP
TSC724	*lyst-1(gk634047); mccIs099; mccEx282*	*mccIs099=aff-1*p::myrmCherry; *mccEx282*=2XFYVE::GFP
TSC725	*skn-1(zj15); nsIs435; mccEx247*	*nsIs435=aff-1*p::myrGFP; *mccEx247*=pPG422
TSC726	*geIs7; mccEx247*	*mccEx247*=pPG422
TSC731	*lyst-1(syb8801); rab-7(mcc38)*	*mcc38*=wrmScarlett::RAB-7
TSC732	*lyst-1(syb8801); nsEx5971*	*nsEx5971=ced-1*p::LAAT-1::mCherry
PHX7213	*sqst-1(syb7213) skn-1(zj15)*	
PHX8801	*lyst-1(syb8801)*	
PHX9268	*lyst-1(syb8801 syb9206 syb9268)*	
OS12750	*ns968; nsIs435*	*ns968: sqst-1* S350N change in exon 2; *nsIs435=aff-1*p::myrGFP
